# Endotype-driven decisions in choosing a biologic for airway diseases

**DOI:** 10.3389/falgy.2025.1754173

**Published:** 2026-01-12

**Authors:** Carmen Panaitescu, Laura Haidar, Maria-Roxana Buzan, Răzvan-Ionuț Zimbru, Valentin-Cristian Iovin, Elena-Larisa Zimbru, Alexandru Lăculiceanu, Ioana Agache

**Affiliations:** 1 Center of Immuno-Physiology and Biotechnologies, Department of Functional Sciences, “Victor Babes” University of Medicine and Pharmacy, Timisoara, Romania; 2 Research Center for Gene and Cellular Therapies in the Treatment of Cancer—OncoGen, “Pius Brinzeu” Timis County Emergency Clinical Hospital, Timisoara, Romania; 3 Allergy & Clinical Immunology, Transylvania University, Brasov, Romania

**Keywords:** asthma, biologics, biomarkers, chronic rhinosinusitis, endotype-driven therapy, precision medicine, type 2 immune response

## Abstract

The increasing availability of biologic therapies for allergic diseases has highlighted the need for more precise, mechanism-based patient selection. Traditional approaches based on disease phenotypes often fall short in predicting therapeutic response. Escalating healthcare costs together with questions about the efficacy of the current management of allergic diseases prompt to a shift toward endotype-driven and biomarker-guided strategies. This review explores the role of endotypes, defined by distinct immunologic, molecular, or cellular mechanisms, in guiding the use of targeted biologics in asthma and in chronic rhinosinusitis with nasal polyps. Endotype classification based on the type 1, type 2 and type 3 immune response is critical for selecting biologics targeting the IgE, IL-5, IL-4/IL-13, or TSLP pathways. The endotype-driven approach in allergic diseases has tremendous potential if incorporated into comprehensive care pathways, with endotype identification playing a key role in the management decision tree, with improved outcomes and greater patient satisfaction. To this purpose this review provides decision algorithms for the endotype-guided approach at the point of care and discusses the unmet needs with potential practical solutions to support a personalized precision approach.

## 1 Introduction

In the evolving landscape of precision medicine, the management of complex immune-mediated diseases such as asthma and chronic rhinosinusitis (CRS) with nasal polyps (CRSwNP) has shifted from generalized treatment strategies to more individualized, mechanism-based approaches. This framework enables clinicians to align therapeutic interventions with the underlying disease biology, thereby enhancing treatment efficacy and patient outcomes.

**Figure 1:**
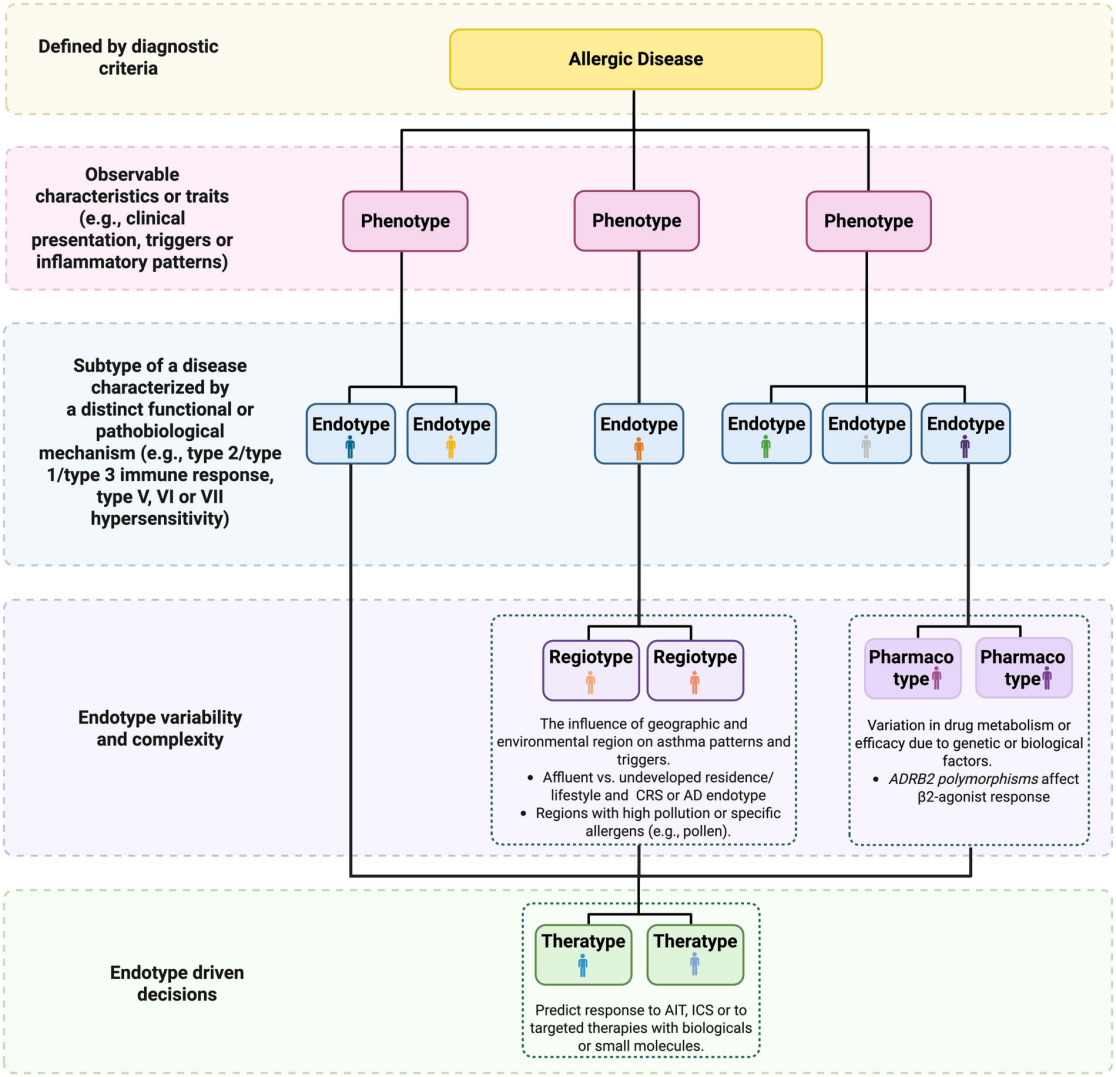
Multidimensional framework for allergic disease stratification. We propose a hierarchical model covering the allergic disease heterogeneity encompassing clinical, biological, environmental and therapeutic dimensions. Phenotypes are defined by observable traits such as symptom patterns and inflammatory profiles, while endotypes are described based on distinct immune mechanisms. Regiotypes reflects environmental and geographical influences, while pharmacotypes explain the variations in drug efficacy due to genetic, anatomic or biological factors. Theratype refers to predicted therapeutic responsiveness based on disease mechanisms. Examples of theratypes include better outcomes with AIT in allergic asthma, with ICS for patients with the T2 gene signature (POSTN, CLCA1, and SERPINB2) or with anti–IL-5 biologics in patients with blood eosinophils > 300/mm^3^. AD, atopic dermatitis; ADRB2, beta-2 adrenergic receptor gene; AIT, allergen immunotherapy; CLCA1, chloride channel regulator 1; CRS, chronic rhinosinusitis; ICS, inhaled corticosteroids; IL, interleukin; POSTN, periostin; SERPINB2, serpin peptidase inhibitor, clade B, member 2; T2, type 2 immune response.

To understand the critical importance of endotype-driven decisions in selecting the biologic for each patient, it is essential to define key terms (Box 1, Figure 1).

Box 1 **Glossary of terms used to define the endotype-driven management of allergic diseases**

**Table:** 

Term	Definition	Comments
Phenotype	refers to the observable characteristics or visible traits of a disease, such as clinical presentation and triggers, inflammatory or molecular pattern, which arise due to interactions between the genome and the environment (1, 2).	While phenotypes provide valuable clinical insights, they may not fully capture the underlying pathophysiological processes Patients with similar phenotypes may have fundamentally different pathophysiological pathways, necessitating different therapeutic approaches.
Endotype	analyze deeper, identifying disease subtypes based on distinct functional or pathobiological mechanism (3, 4).	One of the best examples is type 2 (T2) asthma, a complex endotype characterized by eosinophilic inflammation and responsiveness to specific biologics targeting the interleukin (IL) IL-4 IL-13, IL-5 or immunoglobulin (Ig) E driven pathogenetic pathways Disease endotypes are not fixed entities as they can shift in the same patient following environmental exposure
Regiotype	concept introduced to account for regional variations in disease expression, influenced by environmental factors, lifestyle and/or allergen exposures (5).	Acknowledges that geographic and environmental contexts can modulate disease mechanisms.
Theratype	prediction of response to targeted intervention based on the pathogenetic pathways.	Particularly impactful in managing severe asthma, where biologics are chosen based on the patient's specific endotypic profile (6).
Pharmacotype	accounts for variations in drug efficacy and safety due to genetic or biological factors (7, 8)	

The integration of phenotypic, endotypic, regiotypic, theratypic and pharmacotypic information embodies the essence of precision medicine—delivering the right treatment to the right patient at the right time. As our understanding of disease mechanisms deepens, these classifications will continue to refine therapeutic strategies, ultimately improving patient care and outcomes (Figure 1).

Asthma and CRS are common chronic inflammatory diseases with a well-recognized clinical relationship (CRS is reported in 50% of patients with severe asthma, while asthma is observed in 70% of patients with CRSwNP), thus suggesting a common pathogenesis between these diseases. Both diseases are driven by complex airway epithelial cell and immune cell interactions that occur in response to environmental triggers. Allergens, air pollutants, smoking, and microbial dysbiosis can trigger innate immune responses in epithelial cells, leading to the release of damage-associated molecular patterns (DAMPs) and sustained activation of toll-like receptors (TLRs) and NOD-like receptors (NLRs). The unified airway hypothesis provides a fundamental model to understand the endotype-driven management of asthma and CRS aiming to optimize the diagnosis and treatment.

This review aims to guide the clinician in its management decisions by reviewing each of the components important in developing this therapeutic paradigm and by providing several integrated goals for precision or personalized medicine for asthma and CRS.

## 2 Endotype-driven decisions in asthma

Asthma is a heterogeneous airway disease which comprises a diverse array of pathophysiological mechanisms driving the clinical manifestations (9, 10). Viewed until recently as a single disease, the advances in precision immunology redefined asthma as a complex syndrome characterized by chronic airway inflammation, remodeling and hyperresponsiveness (AHR) driven by several overlapping key pathogenetic pathways (9, 11, 12). Following this concept, asthma management evolved from a one-size-fits-all strategy to precision medicine approaches based on disease endotype (13, 14).

### 2.1 Mechanistic insights—major asthma endotypes

An intricate network of factors that interact at all disease levels drives asthma pathogenesis, from genetic susceptibility and immune system dysregulation to environmental factors' impact, epithelial barrier, mucus production, airway smooth muscle cells (ASM), extracellular matrix, local nerves and blood vessels. The central elements in asthma pathophysiology are chronic airway inflammation and tissue remodeling which can manifest through distinct pathways that define asthma endotypes. Type 2 (T2) immune response is the most studied and is initiated when the airway epithelial cells are fired upon by environmental factors (allergens, pollutants, and viruses) and release signal molecules known as alarmins [thymic stromal lymphopoietin (TSLP), IL (interleukin)-33 and IL-25], which activate innate lymphoid cells (ILC) type 2 (ILC2) and prone dendritic cells (DCs) to a T2 profile. Acting together, ILC2s and DCs recruit and activate T helper (Th) 2 cells with further secretion of IL-4, IL-13 IL-5, IL- 9 and IL-21 effector cytokines (15). IL-5 is involved in the recruitment, maturation and survival of eosinophils, while IL-13 is involved in goblet cell hyperplasia, mucus production and AHR. Together with IL-13, IL-4 induces the switch of B cells into IgE-secreting isotype plasma cells. Mast cells (MCs) and basophils are also recruited and activated (9, 11, 16–18). Beyond the T2 immune-inflammatory pathway, type 1 (T1) or type 3 (Th17) pathways may be activated in asthma. Environmental stimuli (pollutants, tobacco smoke, microbes), induce damage to airway epithelial cells activating the innate lymphoid cells type 3 (ILC3) and Th17 cells which secret pro-neutrophilic cytokines (IL-17 and IL-22). The Th1 pathway can be also involved with activation of ILC1, recruitment of CD4+ Th cells, CD8 + cytotoxic T cells, natural killer (NK) cells, and macrophages, inflammasome activation and production of interferon (IFN)-γ (9, 11, 19, 20).

Based on the predominant immune-inflammatory pathways involved major asthma endotypes include type-2 high asthma (T2 asthma), type-2 low (T1 and T3 asthma), and mixed endotypes (21, 22). These endotypes share a variety of pathophysiological pathways, including genetic and epigenetic, microbiome metabolic, neurogenic, and remodeling pathways (type V, VI and VII asthma) (8, 23). Non-type 2 pathogenetic pathways become noticeable especially in patients with severe T2 asthma, but might be found also in milder cases. A study involving adult patients with stable asthma showed overlapping T2 and non-T2 inflammation in 47.5% of the individuals evaluated (24, 25). A population Th2/Th17 dual-positive asthmatic patients was reported following the examination of bronchoalveolar lavage (BAL) fluid and was associated with more severe airway obstruction and AHR (13, 26, 27). In these patients, partial or no response to T2-targeted biologics can be explained by dominant non-T2 pathways driving the chronic inflammation, AHR and remodeling (24, 25). The contribution of non-T2 pathways within the T2 asthma endotype is supported by the efficacy of tezepelumab, an anti-TSLP monoclonal antibody, proved to reduce the exacerbations of asthma independent of blood eosinophil counts (28).

### 2.2 Biomarkers

Biomarkers play a crucial role in linking asthma endotypes and phenotypes. Among the available biomarkers for T2 immune response in asthma, blood eosinophils, sputum eosinophils, and fractional exhaled nitric oxide (FeNO) are most frequently applied at the point-of care. Each has distinct advantages and limitations that influence their utility for endotype-driven decisions: (1) blood eosinophils are widely available, inexpensive, reproducible, predictive of exacerbation risk and response to anti–IL-5/IL-5R biologics, and they can be monitored longitudinally; however, blood eosinophil counts may be influenced by concomitant corticosteroid treatment, intercurrent infections, and various comorbidities, and the optimal cut-off values for clinical decision-making remain variable across studies (29, 30); (2) sputum eosinophils are considered the gold standard for assessing airway eosinophilic inflammation and correlate closely with disease control and future exacerbations; this parameter is also valuable in guiding inhaled corticosteroids (ICS) titration; however, the method requires specialized laboratory facilities and trained personnel, is technically demanding, and sample quality can be inconsistent, which limits its availability in routine clinical practice and its feasibility for repeated assessments (31–33); (3) FeNO provides a non-invasive, rapid, and patient-friendly assessment of T2 airway inflammation, reflecting IL-4/IL-13 activity, it is particularly useful in monitoring adherence to ICS and in predicting responses to therapies targeting IL-4Rα and IL-13; despite these advantages, FeNO measurements may be affected by age, atopy, smoking status, and respiratory infections, and elevated values are not specific to asthma, being observed in other eosinophilic airway diseases (34, 35). Taken together, these biomarkers provide complementary information. While sputum eosinophils offer the most direct measure of airway inflammation, blood eosinophils and FeNO are more accessible and practical for routine care. An integrated approach that combines these parameters with clinical context remains most informative for tailoring biologic therapy. They should be used within the regiotype and endotype variability framework. For instance, serum periostin, a biomarker commonly used to identify T2 asthma, has differing diagnostic value depending on ethnicity, age or asthma severity. While periostin effectively discriminates asthmatics from non-asthmatics in Japanese and Caucasian populations, it does not perform similarly in Chinese cohorts. Interestingly, Chinese individuals exhibit higher baseline periostin levels than Caucasians, and these levels also vary by sex within the Chinese population (5, 36). Although shown to be related to the T2 pathway in severe asthma periostin proved to be outside of the T2 cluster in patients with moderate asthma (37).

A recent hypothesis proposes that various aspects of asthma treatment response, such as lung function improvement, bronchodilator response, AHR, symptom control, oral corticosteroid (OCS) use, and emergency visits, are influenced by a single, quantitative corticosteroid responsiveness theratype (5). Genetic studies have identified several asthma pharmacotypes based on single nucleotide polymorphisms (SNPs) associated with responses to common asthma treatments (38, 39). Additionally, certain SNPs predict adverse effects of ICS in high doses, such as adrenal suppression (40, 41).

### 2.3 Asthma theratypes

Currently, the endotype-driven strategies for asthma management use several biologic therapies targeting a specific pathogenetic pathway: anti IgE (omalizumab), anti IL-4/IL-13 (dupilumab), anti IL-5 (benralizumab, depemokimab, mepolizumab, reslizumab), TSLP (tezepelumab), IL-33/ST2 (itepekimab, tozorakimab, astegolimab) (14, 42, 43). Currently, 7 biologic agents are approved for asthma by the European Medicines Agency (EMA) and Food and Drug Administration (FDA) (Supplementary Table S1), with the majority targeting T2 asthma (44–46). Only tezepelumab can be used regardless of blood eosinophils.

**Figure 2:**
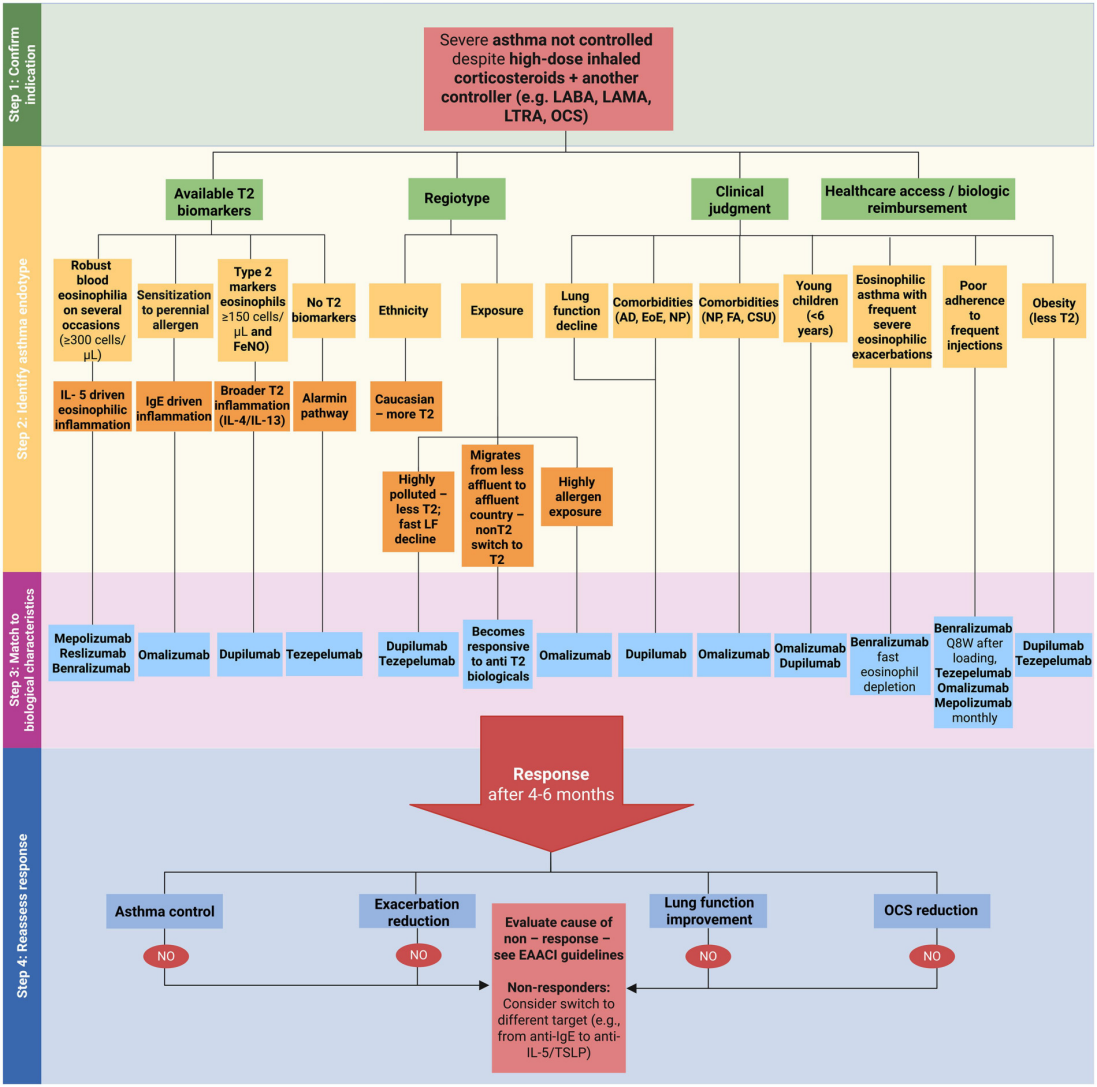
Stepwise algorithm for identifying severe CRSwNP endotypes and selecting targeted biologic therapy. Selection of the biological is guided by T2 biomarkers, regiotype, co-morbidities and treatment accessibility. Treatment response is assessed after 4–6 months using nasal polyp size, olfactory function, quality of life (SNOT-22) and the OCS paring effect. CRSwNP, chronic rhinosinusitis with nasal polyps; IL, interleukin; SNOT-22, 22-item sinonasal outcome test; T2, type 2 immune response.

The major clinical studies on EMA and FDA-approved biologics in asthma are summarised in Supplementary Table S2. Figure 2 presents a stepwise, endotype- and phenotype-driven algorithm to guide the selection of biological treatments in severe asthma, integrating key biomarkers, clinical characteristics, and practical considerations to support personalized therapy decisions.

### 2.4 Defining response

Defining the response to targeted treatment in asthma remains a challenge and involves evaluating various clinical outcomes since there is no universally accepted definition. Recent studies showed that measuring the treatment response is complex and include reduced exacerbation frequency (at least a 50% reduction in the annual number of exacerbations), improved lung function (increase in forced expiratory volume (FEV₁) > 100 mL, OCS and ICS reduction, better symptom control and improved quality of life (QoL). Due to the variability of patient responses, the assessment should be performed using a comprehensive definition of response (47). Real-life data 50% of patients do not meet these criteria and are thus considered non-responders. Furthermore, even among responders, improvements may vary not covering all the clinical end-points (partial responders, dissociated response). The concept of “deep response”, encompassing not only exacerbation reduction but also symptom stability, exercise tolerance, and steroid independence, is gaining traction, particularly in real-world settings. Here, integrating objective measures (exacerbation rate, FEV₁, T2 biomarkers) with patient-reported outcomes (symptom burden, activity limitation, QoL) offers a more comprehensive framework for assessing the therapeutic benefit (48).

As no universally validated criteria for defining optimal response currently exist, several guidelines and consensus documents for biologics in severe asthma recommend incorporating shared decision making as a core element of response assessment (46, 49). The EAACI guidelines advocate for establishing individual, predefined treatment targets in partnership with each patient, based on their priorities and treatment goals, whether these focus on reducing exacerbations, minimizing corticosteroid use, improving QoL, or restoring functional capacity. This patient-centered approach is particularly relevant in a heterogeneous disease such as asthma, where treatment success may vary considerably between individuals.

Finally, the long-term disease modification and prevention of progression (e.g., airway remodeling, lung function decline) deemed as clinical, biological or complete remission is increasingly recognized an attainable target for endotype-driven asthma care (50).

## 3 Endotype-driven decisions in chronic rhinosinusitis

Chronic rhinosinusitis (CRS) is a chronic inflammatory condition involving the nasal passages and sinuses, with multiple contributing factors and with a significant impact both patient's QoL and healthcare system (51–53). For the management at the point-of-care, CRS is broadly classified as CRS with nasal polyps (NP) (CRSwNP) or without (CRSsNP). This binary framework fails to capture the heterogeneity in the disease's pathogenic mechanisms. The recent paradigm shift toward endotype-based classification reflects the pathobiological mechanisms driving chronic inflammation and tissue remodeling and has enabled more precise therapeutic targeting, especially for refractory CRS (54, 55).

### 3.1 Mechanistic insights—major CRS endotypes

CRS is increasingly recognized as a heterogeneous chronic inflammatory disorder rather than a single disease entity, with distinct pathogenic mechanisms driven by several immune-inflammatory pathways and complex remodeling processes. The understanding of CRS pathogenesis has evolved from a purely obstructive-infectious paradigm to a multidimensional model incorporating epithelial barrier dysfunction, type 1, 2 and 3 innate and adaptive immune response, mucus abnormalities, tissue remodeling, and microbial dysbiosis (53, 56).

The current endotypic classification of CRS defines subgroups according to the underlying immune mechanisms, such as T1 (IFN-γ-driven), T2 (IL-4/13, IL-5 or IgE driven) or T3 (IL-17-driven) (53, 55). Though challenging to implement at the point of care, endotyping represents the future of personalized therapy for CRS as it offers deeper insights into pathogenesis and guides targeted interventions (52, 57).

The T2 CRS endotype is the most well-characterized CRS endotype and is usually encountered in Western populations. It is characterized by a robust ILC2- and Th2 driven immune response, in which epithelial-derived cytokines TSLP, IL-33 and IL-25 initiate the T2 downstream signaling cascades (58). Notably, IL-5 and granulocyte-macrophage colony-stimulating factor (GM-CSF) promote eosinophil survival and activation, while IL-13 impairs epithelial barrier integrity by downregulating tight junction proteins such as claudins and occludin (59). T2-driven inflammation is closely associated with specific tissue remodeling patterns. These include basement membrane thickening, subepithelial edema, collagen deposition, and the accumulation of fibrin due to reduced fibrinolytic activity (53, 60). Downregulation of tissue plasminogen activator (tPA) and increased expression of coagulation components contribute to NP formation. Of note, remodeling in T2 CRS is actively modulated by cytokine-induced epithelial-mesenchymal transition (EMT), myofibroblast activation, and angiogenesis (53, 59).

T1 CRS endotype is defined by IFN-γ secretion from Th1 and by involvement of cytotoxic T lymphocytes (CTLs), macrophages and NK cells (56). This endotype is frequently associated with inflammasome activation and epithelial barrier disruption and has been reported to have increased frequency of intracellular pathogens or viral infection. CRSsNP, particularly in Asian populations (58), may exhibit a predominant T1 endotype. Eosinophils are sparse in this endotype. Elevated IL-12, TNF-α, and CXCL10 can serve as biomarkers for this endotype. Histologic biomarkers are epithelial denudation, mononuclear cell infiltration, and increased fibrosis. MMP-1 and IFN-*γ* are inversely correlated with the NP burden, suggesting protective or regulatory roles (55, 61).

T3 CRS endotype is defined by IL-17A and IL-17F secretion from Th17 and ILC3, is usually associated with extracellular bacteria and fungal infections (53). The T3 immune response involves neutrophil recruitment through IL-8, granulocyte colony-stimulating factor (G-CSF), and serum amyloid A (SAA). Recent evidence highlights the involvement of neutrophil extracellular traps (NETs), myeloperoxidase (MPO), and matrix metalloproteinases (MMP) 8, 9 and 3 as key mediators of T3-induced mucosal damage. The remodeling pattern in T3 CRS is distinct from T2 and T1, with excessive fibronectin deposition, basal lamina fragmentation, and increased expression of tissue inhibitors of metalloproteinases (TIMPs) (54, 59).

*Staphylococcus aureus* colonization is particularly important in CRSwNP, where its enterotoxins function as superantigens to amplify Th2 responses, drive local specific IgE synthesis, and impair host immunity. Additionally, *S. aureus*-induced IL-33 and TSLP production sustains chronic inflammation and tissue remodeling (62, 63).

Cluster-based analyses have refined the description of CRS endotypes. A landmark multicenter study in China identified five distinct clusters based on a combination of inflammatory and remodeling markers (56). Clusters 1 and 2 were characterized as non-T2, with high levels of IL-19 and IL-27, suggesting predominant regulatory or homeostatic profiles. IL-27 suppresses both T2 and Th17 responses and was inversely correlated with disease severity and recurrence. Cluster 3 was characterized by a low-T2/high-neutrophilic endotype, with the highest levels of MPO, IL-8, MMPs, and fibronectin. This endotype was associated with severe tissue remodeling and NET formation. Cluster 4 corresponded to moderate T2 inflammation, while cluster 5 exhibited the full T2 signature, including high levels of IL-5, GM-CSF, ECP, total IgE, and EETs (56).

CRS endotypes are influenced by environmental exposures—CRS regiotypes. In Western populations, CRSwNP is predominantly eosinophilic and T2-high, while in East Asia, CRSwNP often exhibits mixed T1/T3 features with low eosinophilia (58). Environmental exposures, microbial patterns, and genetic background likely contribute to these regional variations. For example, cluster 3 CRS in Chinese patients demonstrated significantly higher neutrophilic markers than those reported in European cohorts, emphasizing the need for region-specific endotyping strategies (56, 62, 64).

Clinically, T2 CRS is characterized by bilateral NP and frequent association with comorbidities such as asthma or non-steroidal anti-inflammatory drug (NSAID)-exacerbated respiratory disease (N-ERD). These patients often experience severe symptoms, particularly anosmia and nasal blockage, have high rates of disease recurrence post-surgery or need frequent OCS courses (65, 66). Non-T2 CRS typically presents without NPs and may be associated with pronounced tissue remodeling, including subepithelial fibrosis, goblet cell hyperplasia, and glandular hypertrophy.

### 3.2 Biomarkers

Tissue eosinophilia, increased expression of periostin, eosinophil cationic protein (ECP) and CCL26 in NP tissue in and nasal secretions, the presence of eosinophil extracellular traps (EETs) and increased IgE levels, particularly specific IgE to microbial and self-antigens, serve as a robust signature biomarkers for the T2 CRS endotype (56). Blood eosinophilia and increased total serum IgE are frequently associated with T2 CRS, although the role of total IgE is questionable as it was not related to severity (67). Contrary to FeNO in asthma, nasal NO has not been shown to be helpful to identify the T2 endotype because the main source of production of nasal NO is the sinuses that are closed off when CRS occurs (68). In some more specialized centers tissue and serum periostin levels, IL-5 or IL-33 serum levels can be used (69, 70).

Neutrophils are typically predominant in 50% of patients with CRS without nasal polyps, but also are found to play a role in patients with severe type 2 CRS with nasal polyp disease. Programmed cell death ligand 1, platelet-derived growth factor subunit β [PDGF-β], macrophage inflammatory protein-3b, and PDGF-α were significantly predictive of the surgical outcome in non-eosinophilic CRS (71, 72).

### 3.3 CRS theratypes

The major theratypes of CRS are broadly categorized into T2 and non-T2 (73). This endotypic stratification aligns with principles of precision medicine and is increasingly integrated into clinical decision-making frameworks, such as those outlined in the 2020 European Position Paper on Rhinosinusitis and Nasal Polyps (EPOS 2020) (54, 74–76).

The therapeutic landscape for T2 CRS has been transformed by the introduction of biologic agents targeting key cytokines and immune pathways. The current EMA and FDA-approved biologics for CRSwNP are summarized in Supplementary Table S3; the major clinical studies on EMA-approved biologics in CRSwNP can be found in Supplementary Table S4. Dupilumab, an anti-IL-4Rα monoclonal antibody, has emerged as a first-line biologic for severe CRSwNP, demonstrating robust efficacy in reducing NP size, improving nasal airflow, and decreasing OCS dependence (77). Other approved agents include anti-IL-5 (mepolizumab, reslizumab) and anti-IgE (omalizumab) biologics. For T2 CRS, the integration of biomarker-driven algorithms supports the rational use of biologics, particularly in patients with bilateral NPs, elevated blood eosinophils or OCS dependence. The choice of the tailored intervention depends on biomarker profiles such as tissue eosinophil counts, blood eosinophil levels (≥250/μL), or serum IgE concentrations (≥100 IU/mL) (2, 54, 78).

Management of non-T2 CRS emphasizes infection control, mitigation of environmental exposures, and surgical intervention when appropriate (72, 74, 75). Patients with non-T2 CRS have a suboptimal response to OCS and are generally not candidates for currently available biologic therapies. Instead, long-term low-dose macrolide antibiotics, particularly clarithromycin, have been utilized for their anti-inflammatory and immunomodulatory properties, although high-quality evidence remains limited. Endoscopic sinus surgery (ESS) remains a mainstay for refractory cases, often serving both diagnostic and therapeutic purposes. A pragmatic, three-arm, randomised, placebo-controlled phase 4 trial, evaluated adults with CRS receiving ESS vs. clarithromycin or placebo. ESS proved clinical effectiveness, providing significantly improved disease-specific quality of life at 6 months. Conversely, the trial findings did not support routine long-term use of low-dose clarithromycin (79). Emerging investigational approaches include targeted inhibition of IL-17 and TNF-α pathways, although these are not yet validated for clinical use (5, 72).

**Figure 3:**
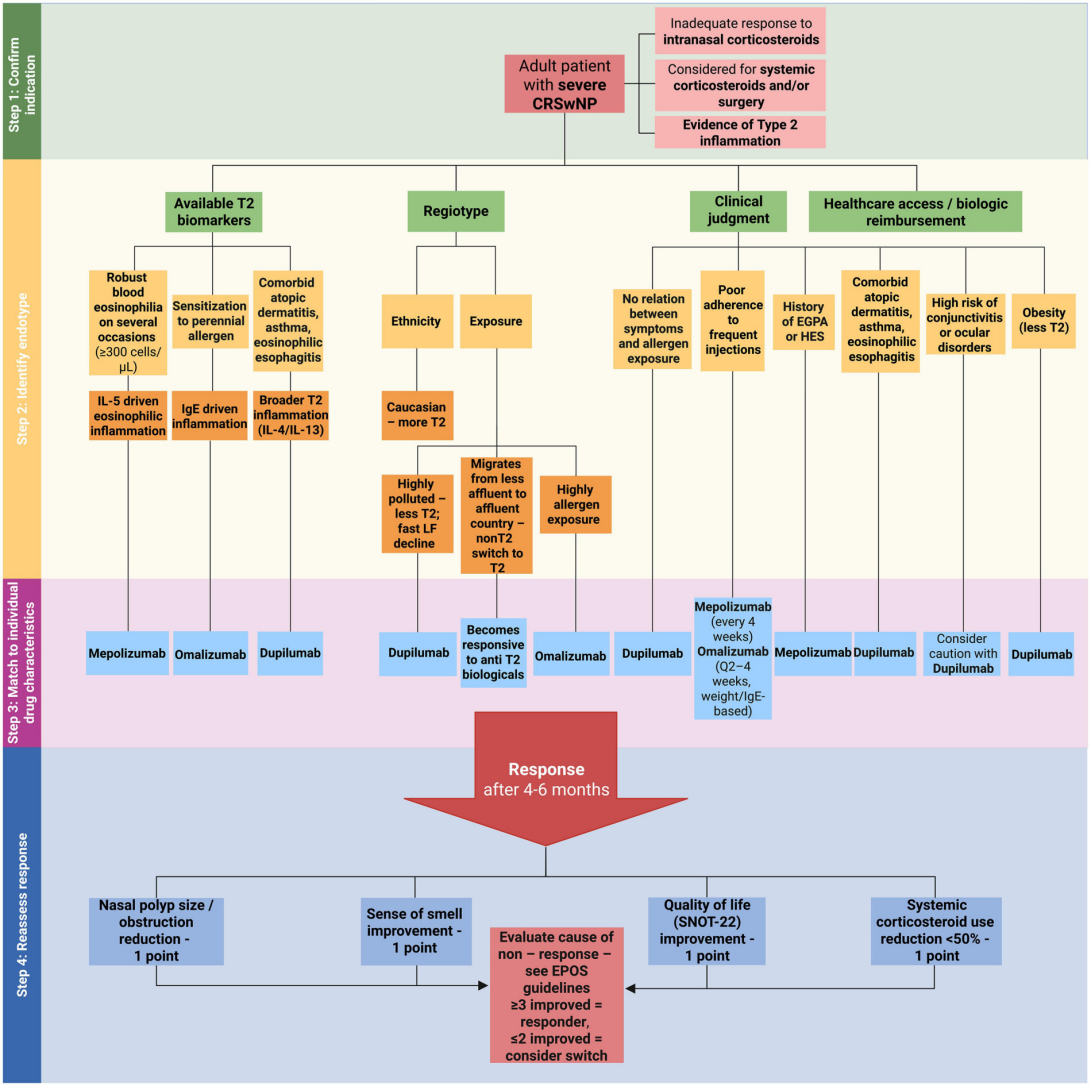
Stepwise algorithm for identifying severe asthma endotypes and guiding biologic therapy selection. We propose an endotype-based approach for managing severe asthma uncontrolled despite high-dose ICS plus another controller (e.g., LABA, LAMA, LTRA or OCS). Biologic selection is guided by T2 biomarkers, regiotype (ethnicity or exposure), clinical judgment and treatment accessibility, linking inflammatory mechanisms with targeted agents such as mepolizumab, reslizumab, benralizumab, omalizumab, dupilumab and tezepelumab. Treatment response is evaluated after 4–6 months, based on achieving asthma control, exacerbation rate, lung function and OCS use reduction. Non-responders should be assessed for adherence, comorbidities or alternative biological targets following EAACI guidelines (46). EAACI, European academy of allergy and clinical immunology; FeNO, fractional exhaled nitric oxide; ICS, inhaled corticosteroids; IL, interleukin; LABA, long-acting β_2_-agonist; LAMA, long-acting muscarinic antagonist; LTRA, leukotriene receptor antagonist; OCS, oral corticosteroids; T2, type 2 inflammation.

Figure 3 presents a stepwise, endotype- and phenotype-driven algorithm to guide the selection of biological treatments in CRSwNP, integrating key biomarkers, clinical characteristics, and practical considerations to support personalized therapy decisions.

### 3.4 Defining response

Defining treatment response in endotype-driven management combines clinical, radiologic, biomarker and patient-reported outcomes.

For T2 CRS response is assessed via nasal endoscopy (Lund-Kennedy/polyp scores), symptom scales (SNOT-22, VAS) and CT imaging (Lund-Mackay). EPOS 2020 outlines five key response criteria: polyp reduction, improved olfaction, fewer steroids, better QoL and comorbidity control, improvement in ≥3 defines success. Biologics like dupilumab show high efficacy in T2-high patients, particularly those with eosinophilia or asthma. Biomarker trends (e.g., decreased IgE, periostin) further guide therapeutic decisions, though transient eosinophilia post-dupilumab does not indicate failure. Non-T2 CRS requires distinct metrics, such as reduction in the neutrophilic markers (IL-8, MMP-9) over 6–12 weeks (54, 80).

Surgical response varies by endotype: T2 CRSwNP often recurs post-ESS without biologics, while non-T2 CRS may reach long-term relief.

CRS control frameworks (e.g., EUFOREA) now emphasize stability, exacerbation rates, and PROMs over isolated metrics (80). Emerging tools, such as AI-guided endoscopy and nasal biomarker signatures, hold the promise for real-time monitoring of the efficacy of targeted interventions.

Given the heterogeneity of CRSwNP and its frequent overlap with comorbid conditions, shared decision making is likewise essential when establishing individualized treatment goals for biologic therapy. Targets such as reduction in NP size, symptom relief (nasal obstruction, loss of smell), decreased need for OCS or surgery, and improved QoL should be defined in partnership with the patient, ensuring that response assessment reflects outcomes meaningful to them (54, 80).

Ultimately, CRS management is evolving toward dynamic, personalized algorithms, integrating biomarkers, biologics and advanced diagnostics for precision care.

## 4 Clinical implementation

The introduction of targeted treatment for asthma and CRS increases the need for biomarkers at the point of care validated for patient selection, prediction of outcomes and monitoring, to allow for an adequate choice of the duration of these costly and long-lasting therapies (81).

For asthma FeNO and blood eosinophils are the only two tests that are currently easily accessible at the point of care. A recent systematic review and meta-analysis confirmed their value for clinical risk stratification and for targeted exacerbation risk reduction (82). Health economics evaluations showed that, given its ability to improve the accurate diagnosis of asthma, monitor treatment response, optimize ICS dosing, and identify patient nonadherence, the FeNO testing strategy may improve the management of asthmatic patients leading to significant savings for the national healthcare systems (83).

To date, no asthma or CRS biomarker has been evaluated in a prospectively collected cohort of the target population using the rigorous “Prospective-specimen-collection, Retrospective-blinded evaluation” (PRoBE) design. Furthermore, the intended use of each biomarker—such as for risk stratification or predicting treatment response—needs to be clearly defined. As depicted in our proposed management algorithm biomarkers should not be used in isolation from clinical characteristics, including age, age of disease onset, the presence of co-morbidities and of the various environmental triggers defining the regiotypes. In the decision-making process, endotypes should be integrated with the assessment of future risk (exacerbation-prone or fast lung function decline) and of the suboptimal response to treatment.

Overall, an adequate balance between guideline informed severity-tailored treatment and precision medicine-based individualized approaches is required.

## 5 Unmet needs, future directions

Despite the transformative impact of biologics in asthma and CRS, several unmet needs remain. First, a significant subset of patients fails to achieve adequate disease control, and even less achieve biological or complete remission, although treated according to current biomarker-driven algorithms. This highlights the complexity and overlap of inflammatory pathways, especially in mixed or non-T2 endotypes, which remain poorly characterized and insufficiently targeted by existing therapies.

Biomarker validation and qualification is another critical gap. Current biomarkers such as blood eosinophils, FeNO, periostin, or total or specific IgE offer only partial predictive value. There is a compelling need for reliable, accessible at the point of care, and dynamic biomarkers (reflecting the variability of disease endotypes in time or with exposure) to better predict targeted treatment response and guide switches or combinations.

Additionally, long-term safety and efficacy data for biologics in diverse populations—including pediatric, elderly, and multimorbid patients— and in real-life, outside strictly controlled clinical trials, are limited. Questions remain about optimal treatment duration and disease modification potential.

One promising avenue for further optimizing endotype-driven therapy is the strategic use of allergen immunotherapy (AIT) in combination with biologics. Although traditionally viewed separately from monoclonal antibody therapies, AIT is itself a biologic intervention, capable of inducing long-term immune tolerance through modulation of allergen-specific immune pathways. Recent studies on using combinations of AIT and biologics for allergies demonstrate that initiating AIT under the protective effect of a biologic (such as omalizumab or tezepelumab) can enhance both the safety and the efficacy of achieving immune tolerance, potentially allowing for sustained disease modification beyond what biologics alone can achieve. Exploring such combined approaches may help expand the therapeutic options for patients with complex allergic endotypes (84, 85).

Furthermore, novel targets should be added, such as enhanced focus on restoring the epithelial barrier, assessment of the mechanisms of asthma and CRS inception, holistic prevention measures following the Planetary Health and the Nature Based Solutions models.

Future directions include expanding indications for existing biologics, developing novel agents targeting upstream drivers (e.g., TSLP, IL-33), and embracing systems biology approaches to refine endotyping. Integrating clinical, molecular, and digital tools into decision-making may usher in a new era of personalized therapy for chronic inflammatory diseases, bridging the gap between mechanism and meaningful clinical outcomes (86).

In the current funding model even simple spirometry or nasal endoscopy access is limited. However, our community should continue to educate and advocate for objective measurements at the point-of care defining the disease endotype, with more sophisticate investigations recommended for severe asthma and CRS patients starting a biological or who exacerbate on biologics. While some barriers remain, the return to investment is high, particularly by reducing exacerbations, emergency departments visits, and poor disease control and by improving QoL (81).

## 6 Conclusion

Endotype-driven strategies represent a paradigm shift in managing asthma, atopic dermatitis, and chronic rhinosinusitis. By aligning therapies with underlying mechanisms, clinicians can personalize care, enhance efficacy, and minimize overtreatment. Continued research into biomarkers and novel targets is essential to refine these approaches and reach broader patient populations.

## References

[1] Funkhouser WK. Pathology: the clinical description of human disease. Mol Pathol. (2009):197–207. 10.1016/B978-0-12-374419-7.00011-1

[2] Kato A Peters AT Stevens WW Schleimer RP Tan BK Kern RC. Endotypes of chronic rhinosinusitis: relationships to disease phenotypes, pathogenesis, clinical findings, and treatment approaches. Allergy. (2022) 77:812–26. 10.1111/all.15074 34473358 PMC9148187

[3] Kuruvilla ME Lee FE-H Lee GB. Understanding asthma phenotypes, endotypes, and mechanisms of disease. Clin Rev Allergy Immunol. (2019) 56:219–33. 10.1007/s12016-018-8712-1 30206782 PMC6411459

[4] Conrad LA Cabana MD Rastogi D. Defining pediatric asthma: phenotypes to endotypes and beyond. Pediatr Res. (2021) 90:45–51. 10.1038/s41390-020-01231-6 33173175 PMC8107196

[5] Agache I Akdis CA. Precision medicine and phenotypes, endotypes, genotypes, regiotypes, and theratypes of allergic diseases. J Clin Invest. (2019) 129:1493–503. 10.1172/JCI124611 30855278 PMC6436902

[6] Porsbjerg CM Townend J Bergeron C Christoff GC Katsoulotos GP Larenas-Linnemann D Association between pre-biologic T2-biomarker combinations and response to biologics in patients with severe asthma. Front Immunol. (2024) 15:1361891. 10.3389/fimmu.2024.1361891 38711495 PMC11070939

[7] Kucuksezer UC Ozdemir C Akdis M Akdis CA. Precision/personalized medicine in allergic diseases and asthma. Arch Immunol Ther Exp. (2018) 66:431–42. 10.1007/s00005-018-0526-6 30251122

[8] Jutel M Agache I Zemelka-Wiacek M Akdis M Chivato T del Giacco S Nomenclature of allergic diseases and hypersensitivity reactions: adapted to modern needs: an EAACI position paper. Allergy. (2023) 78:2851–74. 10.1111/all.15889 37814905

[9] Agache I Akdis CA. Global Atlas of Asthma. 2nd ed. Zurich: European Academy of Allergy and Clinical Immunology (EAACI) (2021). p. 1–344.

[10] GINA-Strategy-Report_2025-WEB-WMS.pdf. Accessed (September 4, 2025).

[11] Russell RJ Brightling CE. Pathogenesis of asthma: implications for precision medicine. Clin Sci. (2017) 131:1723–35. 10.1042/CS20160253 28667070

[12] Agache I Adcock IM Baraldi F Chung KF Eguiluz-Gracia I Johnston SL Personalized therapeutic approaches for asthma. J Allergy Clin Immunol. (2025) 156:503–22. 10.1016/j.jaci.2025.03.025 40203996

[13] Agache I Akdis CA. Endotypes of allergic diseases and asthma: an important step in building blocks for the future of precision medicine. Allergol Int. (2016) 65:243–52. 10.1016/j.alit.2016.04.011 27282212

[14] Agache I Cojanu C Rogozea L. Endotype-driven Approach for Asthma. Implementing Precision Medicine in Best Practices of Chronic Airway Diseases. London: Elsevier (2019). p. 45–9.

[15] Bick F Brenis Gómez CM Lammens I Van Moorleghem J De Wolf C Dupont S IL-2 family cytokines IL-9 and IL-21 differentially regulate innate and adaptive type 2 immunity in asthma. J Allergy Clin Immunol. (2024) 154:1129–45. 10.1016/j.jaci.2024.07.024 39147327 PMC12255943

[16] Coverstone AM Seibold MA Peters MC. Diagnosis and management of T2-high asthma. J Allergy Clin Immunol Pract. (2020) 8:442–50. 10.1016/j.jaip.2019.11.020 32037108

[17] Hammad H Lambrecht BN. The basic immunology of asthma. Cell. (2021) 184:1469–85. 10.1016/j.cell.2021.02.016 33711259

[18] Whetstone CE Amer R Maqbool S Javed T Gauvreau GM. Pathobiology and regulation of eosinophils, mast cells, and basophils in allergic asthma. Immunol Rev. (2025) 331:e70018. 10.1111/imr.70018 40235366 PMC12001016

[19] Peri F Amaddeo A Badina L Maschio M Barbi E Ghirardo S. T2-low asthma: a discussed but still orphan disease. Biomedicines. (2023) 11:1226. 10.3390/biomedicines11041226 37189844 PMC10136127

[20] Sze E Bhalla A Nair P. Mechanisms and therapeutic strategies for non-T2 asthma. Allergy. (2020) 75:311–25. 10.1111/all.13985 31309578

[21] Rogliani P Calzetta L Matera MG Laitano R Ritondo BL Hanania NA Severe asthma and biological therapy: when, which, and for whom. Pulm Ther. (2020) 6:47–66. 10.1007/s41030-019-00109-1 32048241 PMC7229123

[22] Bagnasco D Testino E Nicola S Melissari L Russo M Canevari RF Specific therapy for T2 asthma. J Pers Med. (2022) 12:593. 10.3390/jpm12040593 35455709 PMC9031027

[23] Agache I Cojanu C Laculiceanu A Rogozea L. Critical points on the use of biologicals in allergic diseases and asthma. Allergy Asthma Immunol Res. (2020) 12(1):24–41. 10.4168/aair.2020.12.1.24 31743962 PMC6875478

[24] Han YY Zhang X Wang J Wang G Oliver BG Zhang HP Multidimensional assessment of asthma identifies clinically relevant phenotype overlap: a cross-sectional study. J Allergy Clin Immunol Pract. (2021) 9:349–362.e18. 10.1016/j.jaip.2020.07.048 32791248

[25] Seys SF Long MB. The quest for biomarkers in asthma: challenging the T2 versus non-T2 paradigm. Eur Respir J. (2022) 59:2102669. 10.1183/13993003.02669-2021 35177484

[26] Cosmi L Maggi L Santarlasci V Capone M Cardilicchia E Frosali F Identification of a novel subset of human circulating memory CD4+ T cells that produce both IL-17A and IL-4. J Allergy Clin Immunol. (2010) 125:222–230.e4. 10.1016/j.jaci.2009.10.012 20109749

[27] Irvin C Zafar I Good J Rollins D Christianson C Gorska MM Increased frequency of dual-positive T_H_2/T_H_17 cells in bronchoalveolar lavage fluid characterizes a population of patients with severe asthma. J Allergy Clin Immunol. (2014) 134:1175–1186.e7. 10.1016/j.jaci.2014.05.038 25042748 PMC4254017

[28] Corren J Parnes JR Wang L Mo M Roseti SL Griffiths JM Tezepelumab in adults with uncontrolled asthma. N Engl J Med. (2017) 377:936–46. 10.1056/NEJMoa1704064 28877011

[29] Porpodis K Tsiouprou I Apostolopoulos A Ntontsi P Fouka E Papakosta D Eosinophilic asthma, phenotypes-endotypes and current biomarkers of choice. JPM. (2022) 12:1093. 10.3390/jpm12071093 35887589 PMC9316404

[30] Mathioudakis AG Bate S Sivapalan P Jensen J-US Singh D Vestbo J. Rethinking blood eosinophils for assessing inhaled corticosteroids response in COPD. Chest. (2024) 166:987–97. 10.1016/j.chest.2024.06.3790 38992490 PMC11562658

[31] Mangattu Parambil PB Mohapatra AK Behera D Subhankar S Jagaty SK Singh P. Determination of sputum eosinophil count and serum absolute eosinophil count in patients with bronchial asthma and its correlation with disease severity and response to treatment. J Family Med Prim Care. (2023) 12:2053–7. 10.4103/jfmpc.jfmpc_487_23 38024908 PMC10657076

[32] Ali MM Wolfe MG Mukherjee M Radford K Patel Z White D A sputum bioassay for airway eosinophilia using an eosinophil peroxidase aptamer. Sci Rep. (2022) 12:22476. 10.1038/s41598-022-26949-7 36577785 PMC9797489

[33] Mukherjee M Bernaola J Nolasco S Kjarsgaard M Xie Y Radford K Up-dosing of reslizumab in severe asthmatics with persistent sputum eosinophilia: a feasibility study. Allergy. (2025) 80:605–8. 10.1111/all.16322 39286951 PMC11804305

[34] Maniscalco M Fuschillo S Mormile I Detoraki A Sarnelli G Paulis AD Exhaled nitric oxide as biomarker of type 2 diseases. Cells. (2023) 12:2518. 10.3390/cells12212518 37947596 PMC10649630

[35] Ragnoli B Radaeli A Pochetti P Kette S Morjaria J Malerba M. Fractional nitric oxide measurement in exhaled air (FeNO): perspectives in the management of respiratory diseases. Ther Adv Chronic Dis. (2023) 14:20406223231190480. 10.1177/20406223231190480 37538344 PMC10395178

[36] Tan E Varughese R Semprini R Montgomery B Holweg C Olsson J Serum periostin levels in adults of Chinese descent: an observational study. Allergy Asthma Clin Immunol. (2018) 14:1–9. 10.1186/s13223-018-0312-3 30574168 PMC6299536

[37] Agache I Strasser DS Klenk A Agache C Farine H Ciobanu C Serum IL -5 and IL -13 consistently serve as the best predictors for the blood eosinophilia phenotype in adult asthmatics. Allergy. (2016) 71:1192–202. 10.1111/all.12906 27060452

[38] Keskin O Uluca U Keskin M Gogebakan B Kucukosmanoglu E Ozkars MY The efficacy of single-high dose inhaled corticosteroid versus oral prednisone treatment on exhaled leukotriene and 8-isoprostane levels in mild to moderate asthmatic children with asthma exacerbation. Allergol Immunopathol. (2016) 44:138–48. 10.1016/j.aller.2015.05.006 26318413

[39] Keskin O Uluca Ü Birben E Coşkun Y Ozkars MY Keskin M Genetic associations of the response to inhaled corticosteroids in children during an asthma exacerbation. Pediatr Allergy Immunol. (2016) 27:507–13. 10.1111/pai.12566 27003716

[40] Hawcutt DB Francis B Pirmohamed M. Adrenal suppression with inhaled corticosteroids: the seed and the soil–Authors’ reply. Lancet Respir Med. (2018) 6:e20. 10.1016/S2213-2600(18)30149-8 29856322

[41] Hawcutt DB Francis B Carr DF Jorgensen AL Yin P Wallin N Susceptibility to corticosteroid-induced adrenal suppression: a genome-wide association study. Lancet Respir Med. (2018) 6:442–50. 10.1016/S2213-2600(18)30058-4 29551627 PMC5971210

[42] Agache I Sugita K Morita H Akdis M Akdis CA. The complex type 2 endotype in allergy and asthma: from laboratory to bedside. Curr Allergy Asthma Rep. (2015) 15:1–8. 10.1007/s11882-015-0529-x 26141574

[43] Agache IO. Endotype driven treatment of asthma: endotypes and asthma treatment. Curr Treat Options Allergy. (2014) 1:198–212. 10.1007/s40521-014-0014-0

[44] Cazzola M Ora J Cavalli F Rogliani P Matera MG. Treatable mechanisms in asthma. Mol Diagn Ther. (2021) 25:111–21. 10.1007/s40291-021-00514-w 33570719 PMC7956930

[45] Oberle AJ Abbas F Adrish M Agache I Conroy M Coz A Biologic management in severe asthma for adults: an American college of chest physicians clinical practice guideline. Chest. (2025):S0012-3692(25)05380-2. 10.1016/j.chest.2025.08.042 41005695

[46] Agache I Akdis CA Akdis M Canonica GW Casale T Chivato T EAACI biologicals guidelines—recommendations for severe asthma. Allergy. (2021) 76:14–44. 10.1111/all.14425 32484954

[47] Hansen S Baastrup Søndergaard M Bülow v Bjerrum A Schmid AS Rasmussen J Clinical response and remission in patients with severe asthma treated with biologic therapies. Chest. (2024) 165:253–66. 10.1016/j.chest.2023.10.046 37925144

[48] Panagiotou M Koulouris NG Rovina N. Physical activity: a missing link in asthma care. J Clin Med. (2020) 9:706. 10.3390/jcm9030706 32150999 PMC7141291

[49] Papadopoulos NG Barnes P Canonica GW Gaga M Heaney L Menzies-Gow A The evolving algorithm of biological selection in severe asthma. Allergy. (2020) 75:1555–63. 10.1111/all.14256 32124991

[50] Mailhot-Larouche S Celis-Preciado C Heaney LG Couillard S. Identifying super-responders: a review of the road to asthma remission. Ann Allergy Asthma Immunol. (2025) 134:31–45. 10.1016/j.anai.2024.09.023 39383944

[51] Seah JJ Thong M Wang DY. The diagnostic and prognostic role of biomarkers in chronic rhinosinusitis. Diagnostics. (2023) 13:715. 10.3390/diagnostics13040715 36832203 PMC9955000

[52] Brzost J Czerwaty K Dżaman K Ludwig N Piszczatowska K Szczepański M. Perspectives in therapy of chronic rhinosinusitis. Diagnostics. (2022) 12:2301. 10.3390/diagnostics12102301 36291990 PMC9600269

[53] Toppila-Salmi S Reitsma S Hox V Gane S Eguiluz-Gracia I Shamji M Endotyping in chronic rhinosinusitis—an EAACI task force report. Allergy. (2025) 80:132–47. 10.1111/all.16418 39641584 PMC11724251

[54] Fokkens WJ Lund VJ Hopkins C Hellings PW Kern R Reitsma S European position paper on rhinosinusitis and nasal polyps 2020. Rhin. (2020) 0:1–464. 10.4193/Rhin20.600 32077450

[55] Locatello LG Tonon S Mele V Santini S Miani C Pucillo CEM. Update on the biological and clinical relevance of mast cells in chronic rhinosinusitis with nasal polyps. Biomedicines. (2024) 12:2647. 10.3390/biomedicines12112647 39595211 PMC11592168

[56] Wang X Sima Y Zhao Y Zhang N Zheng M Du K Endotypes of chronic rhinosinusitis based on inflammatory and remodeling factors. J Allergy Clin Immunol. (2023) 151:458–68. 10.1016/j.jaci.2022.10.010 36272582

[57] Fadda GL Rustichelli C Soccal S Moglio S Serrone A Bertolini F Dupilumab in the treatment of severe uncontrolled chronic rhinosinusitis with nasal polyps (CRSwNP) and comorbid asthma—a multidisciplinary monocentric real-life study. Biomedicines. (2025) 13:501. 10.3390/biomedicines13020501 40002914 PMC11853246

[58] Zhang Y Gevaert E Lou H Wang X Zhang L Bachert C Chronic rhinosinusitis in Asia. J Allergy Clin Immunol. (2017) 140:1230–9. 10.1016/j.jaci.2017.09.009 28987810

[59] Czerwaty K Piszczatowska K Brzost J Ludwig N Szczepański MJ Dżaman K. Immunological aspects of chronic rhinosinusitis. Diagnostics. (2022) 12:2361. 10.3390/diagnostics12102361 36292050 PMC9600442

[60] Lee K Tai J Lee SH Kim TH. Advances in the knowledge of the underlying airway remodeling mechanisms in chronic rhinosinusitis based on the endotypes: a review. Int J Mol Sci. (2021) 22:910. 10.3390/ijms22020910 33477617 PMC7831322

[61] Cao P-P Wang Z-C Schleimer RP Liu Z. Pathophysiologic mechanisms of chronic rhinosinusitis and their roles in emerging disease endotypes. Ann Allergy Asthma Immunol. (2019) 122:33–40. 10.1016/j.anai.2018.10.014 30326322 PMC6309633

[62] Kim S-D Cho K-S. Treatment strategy of uncontrolled chronic rhinosinusitis with nasal polyps: a review of recent evidence. Int J Mol Sci. (2023) 24:5015. 10.3390/ijms24055015 36902445 PMC10002552

[63] Xu Z Yan J Wen W Zhang N Bachert C. Pathophysiology and management of Staphylococcus aureus in nasal polyp disease. Expert Rev Clin Immunol. (2023) 19(8):981–92. 10.1080/1744666X.2023.2233700 37409375

[64] Sitzia E Santarsiero S Marini G Majo F De Vincentiis M Cristalli G Endotypes of nasal polyps in children: a multidisciplinary approach. J Pers Med. (2023) 13:707. 10.3390/jpm13050707 37240876 PMC10219557

[65] Xu X Reitsma S Wang DY Fokkens WJ. Highlights in the advances of chronic rhinosinusitis. Allergy. (2021) 76:3349–58. 10.1111/all.14892 33948955

[66] Bachert C Bhattacharyya N Desrosiers M Khan AH. Burden of disease in chronic rhinosinusitis with nasal polyps. J Asthma Allergy. (2021) 14:127–34. 10.2147/JAA.S290424 33603409 PMC7886239

[67] Cavaliere C Seys SF De Kinderen J Bettio G Andrianakis A Alobid I Characterization of chronic rhinosinusitis patients based on markers of type 2 inflammation: findings from the European CRS outcome registry (CHRINOSOR). Clin Transl Allergy. (2025) 15:e70095. 10.1002/clt2.70095 40887890 PMC12399834

[68] Rimmer J Hellings P Lund VJ Alobid I Beale T Dassi C European Position paper on diagnostic tools in rhinology. Rhinology. (2019) 57(S28):1–41. 10.4193/Rhin19.410 31376816

[69] Danielides G Lygeros S Kanakis M Naxakis S. Periostin as a biomarker in chronic rhinosinusitis: a contemporary systematic review. Int Forum Allergy Rhinol. (2022) 12(12):1535–50. 10.1002/alr.23018 35514144

[70] Zielińska-Bliźniewska H Paprocka-Zjawiona M Merecz-Sadowska A Zajdel R Bliźniewska-Kowalska K Malinowska K. Serum IL-5, POSTN and IL-33 levels in chronic rhinosinusitis with nasal polyposis correlate with clinical severity. BMC Immunol. (2022) 23(1):33. 10.1186/s12865-022-00507-2 35752781 PMC9233770

[71] Li Z Lu T Sun L Hou Y Chen C Lai S Factors for predicting the outcome of surgery for non-eosinophilic chronic rhinosinusitis with nasal polyps. Ann Allergy Asthma Immunol. (2024) 133(5):559–67. 10.1016/j.anai.2024.05.023 38880209

[72] Cui N Zhu X Zhao C Meng C Sha J Zhu D. A decade of pathogenesis advances in non-type 2 inflammatory endotypes in chronic rhinosinusitis: 2012–2022. Int Arch Allergy Immunol. (2023) 184:1237–53. 10.1159/000532067 37722364

[73] Tomassen P Vandeplas G Van Zele T Cardell L-O Arebro J Olze H Inflammatory endotypes of chronic rhinosinusitis based on cluster analysis of biomarkers. J Allergy Clin Immunol. (2016) 137:1449–1456.e4. 10.1016/j.jaci.2015.12.1324 26949058

[74] Agache I Song Y Alonso-Coello P Vogel Y Rocha C Solà I Efficacy and safety of treatment with biologicals for severe chronic rhinosinusitis with nasal polyps: a systematic review for the EAACI guidelines. Allergy. (2021) 76(8):2337–53. 10.1111/all.14809 33683704

[75] Cavaliere C Masieri S Begvarfaj E Loperfido A Baroncelli S Cascone F Long-term perspectives on chronic rhinosinusitis with nasal polyps: evaluating recurrence rates after functional endoscopic sinus surgery in the biologics era—a 5-year follow-up study. J Pers Med. (2024) 14:297. 10.3390/jpm14030297 38541039 PMC10971479

[76] Grayson JW Cavada M Harvey RJ. Clinically relevant phenotypes in chronic rhinosinusitis. J Otolaryngol Head Neck Surg. (2019) 48:23. 10.1186/s40463-019-0350-y 31142355 PMC6542143

[77] Bachert C Han JK Desrosiers M Hellings PW Amin N Lee SE Efficacy and safety of dupilumab in patients with severe chronic rhinosinusitis with nasal polyps (LIBERTY NP SINUS-24 and LIBERTY NP SINUS-52): results from two multicentre, randomised, double-blind, placebo-controlled, parallel-group phase 3 trials. Lancet. (2019) 394:1638–50. 10.1016/S0140-6736(19)31881-1 31543428

[78] Bayar Muluk N Cingi C Scadding GK Scadding G. Chronic rhinosinusitis—could phenotyping or endotyping aid therapy? Am J Rhinol Allergy. (2019) 33:83–93. 10.1177/1945892418807590 30353741

[79] Philpott C Beard DJ Saeedi E Cook JA Jones S Clarke CS The clinical effectiveness of clarithromycin versus endoscopic sinus surgery for adults with chronic rhinosinusitis with and without nasal polyps (MACRO): a pragmatic, multicentre, three-arm, randomised, placebo-controlled phase 4 trial. Lancet. (2025) 406(10506):926–39. 10.1016/S0140-6736(25)01248-6 40885584

[80] Fokkens WJ De Corso E Backer V Bernal-Sprekelsen M Bjermer L Von Buchwald C EPOS2020/EUFOREA Expert opinion on defining disease states and therapeutic goals in CRSwNP. Rhin. (2024) 0:0. 10.4193/Rhin23.415 38217529

[81] Agache I Zemelka-Wiacek M Gawlik R Georas SN Jahnz-Różyk K Kupczyk M Targeted treatment in asthma—opportunities and challenges. J Allergy Clin Immunol Pract. (2025):S2213-2198(25)01128-6. 10.1016/j.jaip.2025.11.023 41314466

[82] Meulmeester FL Mailhot-Larouche S Celis-Preciado C Lemaire-Paquette S Ramakrishnan S Wechsler ME Inflammatory and clinical risk factors for asthma attacks (ORACLE2): a patient-level meta-analysis of control groups of 22 randomised trials. Lancet Respir Med. (2025) 13(6):505–16. 10.1016/S2213-2600(25)00037-2 40215991 PMC12117016

[83] Arnold RJG Layton A Massanari M. Cost impact of monitoring exhaled nitric oxide in asthma management. Allergy Asthma Proc. (2018) 39(5):338–34. 10.2500/aap.2018.39.4165 30103840

[84] Olivieri B Günaydın FE Corren J Senna G Durham SR. The combination of allergen immunotherapy and biologics for inhalant allergies: exploring the synergy. Ann Allergy Asthma Immunol. (2025) 134:385–95. 10.1016/j.anai.2024.06.016 38897405

[85] Corren J Larson D Altman MC Segnitz RM Avila PC Greenberger PA Effects of combination treatment with tezepelumab and allergen immunotherapy on nasal responses to allergen: a randomized controlled trial. J Allergy Clin Immunol. (2023) 151:192–201. 10.1016/j.jaci.2022.08.029 36223848 PMC12205947

[86] Staudacher AG Peters AT Kato A Stevens WW. Use of endotypes, phenotypes, and inflammatory markers to guide treatment decisions in chronic rhinosinusitis. Ann Allergy Asthma Immunol. (2020) 124:318–25. 10.1016/j.anai.2020.01.013 32007571 PMC7192133

