# Supplementary material for: Endotype-driven decisions in choosing a biologic for airway diseases
Source: Front Allergy. 2026 Jan 12;6:1754173. doi: 10.3389/falgy.2025.1754173 (PMC12832300; doi:10.3389/falgy.2025.1754173)
Supplement: Supplementary file 1 [file Table1.docx]

**Table legends**

**Table S1.** Biologic therapies for asthma approved by EMA.

**Table S2** Major clinical studies on EMA-approved biologics in asthma.

**Table S3** Biologic therapies for chronic rhinosinusitis with nasal polyps (CRSwNP) approved by EMA.

**Table S4** Major clinical studies on EMA-approved biologics in chronic rhinosinusitis with nasal polyps.

Table S1. Biologic therapies for asthma approved by EMA.

| **Medicine** | **Active substance** | **Mechanism of action** | **Biomarkers for the theratype** | **Patient population** | **Year of EMA approval** | **Dosing regimen** | **Safety profile / Common side effects** | **Formulation & presentation** | **Other indications** |
| --- | --- | --- | --- | --- | --- | --- | --- | --- | --- |
| **Xolair (1)** | Omalizumab | IgG1 monoclonal antibody  Binds free IgE  Inhibits IgE binding to both its high and low-affinity receptors  Inhibits mast cell activation | Blood eosinophils (secondary predictor for CRSwNP overlap), FeNO, childhood-onset asthma, clinical history suggesting allergen-driven symptoms, | Severe allergic asthma (≥6 years) with positive skin test to perennial aeroallergen and uncontrolled on high-dose ICS+LABA | 2005 | Subcutaneous, every 2–4 weeks (dose based on weight and total IgE level) | Injection-site reactions, headache, anaphylaxis risk (rare) | Prefilled syringe/pen (75 mg solution) | CRSwNP  Chronic spontaneous urticaria  Food allergy (FDA) |
| **Nucala (2)** | Mepolizumab | Fully-humanized monoclonal IgG1 kappa  Prevents IL-5 binding to IL-5Rα; Inhibits maturation, activation, proliferation and recruitment of eosinophils | Blood eosinophils, number of severe exacerbations in the previous year, adult-onset asthma, nasal polyps, maintenance OCS at baseline, lung function | Severe eosinophilic asthma (≥6 years) uncontrolled on high-dose ICS+LABA | 2015 | Subcutaneous, 100 mg every 4 weeks | Headache, injection site reactions, back pain, fatigue | Prefilled syringe (40/100 mg solution) | Eosinophilic granulomatosis with polyangiitis (EGPA), hypereosinophilic syndrome (HES) |
| **Cinqaero (3)** | Reslizumab | Humanized IgG4 monoclonal antibody  Prevents IL-5 binding to IL-5Rα; Inhibits maturation, activation, proliferation and recruitment of eosinophils | Blood eosinophils, number of severe exacerbations in the previous year, adult-onset asthma, nasal polyps, maintenance OCS at baseline, lung function | Severe eosinophilic asthma (adults) uncontrolled on high-dose ICS+LABA | 2016 | IV, 3 mg/kg body weight every 4 weeks | Oropharyngeal pain, increased CPK, myalgia, rare anaphylaxis | IV infusion (10 mg/mL) | None |
| **Fasenra (4)** | Benralizumab | Humanized IgG1k monoclonal antibody  Targets IL-5Rα and induces ADCC Depletes the eosinophils and reduces the number of basophils | Blood eosinophils, number of severe exacerbations in the previous year, adult-onset asthma, nasal polyps, maintenance OCS at baseline, lung function | Severe eosinophilic asthma (≥12 years) uncontrolled on high-dose ICS+LABA | 2018 | Subcutaneous, 30 mg every 4 weeks (first 3 doses), then every 8 weeks | Headache, pharyngitis, injection site reactions | Prefilled syringe/pen (30 mg solution) | Eosinophilic granulomatosis with polyangiitis |
| **Dupixent (5)** | Dupilumab | Humanized IgG4 monoclonal antibody  Targets IL-4Rα, shared by IL-4 and IL-13; blocks IL-4/IL-13 signaling | FeNO  Blood eosinophils, | Severe T2-high asthma (≥12 years) with eosinophilia or OCS-dependent | 2017 | Subcutaneous, 200-300 mg every 2 weeks | Injection site reactions, conjunctivitis, oropharyngeal pain | Prefilled syringe/pen (200 mg/300 mg) | AD  CRSwNP  Prurigo nodularis  Eosinophilc esophagitis  COPD |
| **Tezspire (6)** | Tezepelumab | Human immunoglobulin G2λ  Blocks upstream the epithelial derived cytokine TSLP | Blood eosinophils, FeNO | Severe asthma (≥12 years) regardless of eosinophil levels | 2021 | Subcutaneous, 210 mg every 4 weeks | Injection site reactions, pharyngitis, arthralgia | Prefilled syringe/pen (210 mg solution) | None |
| **Omlyclo (7)** | Omalizumab biosimilar | Humanised monoclonal antibody manufactured by recombinant DNA technology targeting IgE | Same as Xolair | Severe allergic asthma (≥6 years) with positive skin test to perennial aeroallergen and uncontrolled on high-dose ICS+LABA | 2024 | Subcutaneous, every 2–4 weeks (dose based on weight/IgE) | Injection-site reactions, headache, anaphylaxis risk (rare) | Prefilled syringe/pen (75 mg solution) | CRSwNP |

*Abbreviations: COPD: chronic obstructive lung disease; CRSwNP: chronic rhinosinusitis with nasal polyps; EMA: European Medicines Agency; FDA: United States Food and Drug Administration; FeNO: fractional exhaled nitric oxide; ICS: inhaled corticosteroids; IgE: immunoglobulin E; LABA: long-acting beta agonist; OCS: oral corticosteroids; TSLP: thymic stromal lymphopoietin.*

Notes regarding biologics available for asthma:

- **Self-administration suitability:** almost all biologics are designed for self-administration using prefilled syringes or pens, with appropriate patient training, with the exception of reslizumab (Cinqaero), which is an intravenous infusion not suitable for self-administration
- **Pregnancy and lactation data:** for all agents, safety data for pregnant or lactating women are limited. Animal studies have not shown harm, but human IgG is known to cross the placenta and is present in breast milk. Use during pregnancy or lactation should be considered with caution and close follow-up.
- **Storage and handling: a**ll biologics require refrigeration at 2–8°C and should not be frozen. Some allow limited room temperature storage (e.g., Dupilumab and Benralizumab up to 14 days at ≤25°C; Tezepelumab up to 30 days) (4–6).
- **Discontinuation criteria: g**enerally recommended to discontinue if no clinical benefit is achieved after 16–24 weeks of therapy (expert opinion based). Immediate discontinuation if severe hypersensitivity or anaphylaxis occurs.
- **Immunogenicity data:** all biologics exhibit low immunogenicity rates (less than 5% of patients develop anti-drug antibodies (ADA)). Neutralizing ADA are rare and have not been associated with loss of efficacy or increased adverse events.

Table S2 Major clinical studies on EMA-approved biologics in asthma.

| **Biological** | **Study** | **Duration** | **Patients (n)** | **Primary endpoint** | **Primary outcome** | **Secondary outcome** | **Publication year** | **Phenotype** |
| --- | --- | --- | --- | --- | --- | --- | --- | --- |
| Omalizumab | INNOVATE (s.c. omalizumab versus placebo to high-dose ICS+LABA±OCS) (8) | 28 weeks | 419 | The rate of clinically significant asthma exacerbations | Relative risk reduction in clinically significant asthma exacerbation rate by 26% | Severe exacerbations were reduced by 50%, and emergency room visits decreased by 44% compared to placebo. The mean AQLQ score improved by 0.45 points more than placebo, approaching the minimal clinically important difference (MCID) of 0.5 points. Global Evaluation of Treatment Effectiveness (GETE) showed clinical improvement | 2004 | Severe persistent asthma with positive skin-prick test, total IgE levels of 30–700 IU/mL |
| Omalizumab | EXTRA (NCT00314575) (s.c.versus placebo to medium- to high-dose ICS) (9) | 48 weeks | 848 | The rate of protocol-defined asthma exacerbations during the 48-week treatment period | Relative risk reduction in protocol defined asthma exacerbation rate by 25% | Improved mean AQLQ scores from baseline by 0.23 points greater than placebo. Improved mean TASS | 2011 | Severe allergic asthma, confirmed by positive skin-prick test or in vitro RAST, with serum IgE levels between 30 and 700 IU/mL and a baseline FEV₁ between 40% and 80% of predicted. Patients receiving maintenance oral corticosteroids (mOCS) were also eligible for inclusion |
| Omalizumab | *Post Hoc* Analysis of EXTRA (NCT00314574)(s.c. versus placebo to high-dose ICS and LABA±additional controller medications)(10) | 48 weeks | 813 | The rate of exacerbations and change from baseline in prebronchodilator FEV1 | In patients with high bronchodilator responsiveness (BDR ≥12%), omalizumab reduced exacerbations by: 59.8% in those with FAO⁺ and 44,3% in those FAO⁻.  In low BDR (<12%) subgroups, omalizumab did not significantly reduce exacerbation rates. | The mean relative FEV₁ improvement in the responsive phenotype (FAO⁻ / high BDR) was roughly +3% to +7% of predicted values, corresponding to an absolute increase of 65–236 mL compared with placebo. In all other subgroups, the change was negligible (<1–2%) and not statistically significant. | 2022 | Severe, persistent allergic asthma for >1 year before screening. Patients were also required to have baseline prebronchodilator FEV1 of 40%-80% of predicted values, serum IgE level of 30-700 IU/mL, and body weight of 30-150 kg |
| Mepolizumab | DREAM (NCT01000506) (i.v.mepolizumab versus placebo to high-dose ICS±LABA±OCS) (11) | 52 weeks | 621 | Annual rate of clinical asthma exacerbations | Relative risk reduction in annual AER by 48% at 75mg, 39% at 250mg and 52% at 750mg dose | Delayed time to first exacerbations versus placebo (HR 0.45 with 75 mg, 0.60 with 250 mg, and 0.46 with 750 mg versus placebo). Change in ACQ from baseline (from −0.75 to − 0.87 versus −0.59), not significant. Change in AQLQ from baseline (0.77–0.93 versus 0.71), not significant | 2012 | Severe eosinophilic asthma, ⩾2 exacerbations, evidence of eosinophilic inflammation: sputum eosinophils ⩾3%, FENO ⩾50 ppb, peripheral blood eosinophils ⩾300 cells·µL−1 Patients on mOCS included |
| Mepolizumab | MENSA (NCT01691521) (i.v.or s.c. mepolizumab versus add-on placebo) (12) | 32 weeks | 576 | Annual rate of clinical asthma exacerbations | Relative risk reduction in clinically significant exacerbation by 47% in the i.v. group and by 53%in the s.c. group | Relative risk reduction in rate of exacerbations leading to hospitalisation or ER visits by 32% in the i.v. and 61% in the s.c. group. Improvement in SGRQ score from baseline by 6.4 and 7.0 points greater than in placebo (MCID, 4 points) Improvement in ACQ-5 from baseline by 0.42–0.44 points greater than in placebo (MCID, 0.5 points) | 2014 | Severe eosinophilic asthma with ⩾2 exacerbations, elevated eosinophils ⩾150 cells·µL−1 at screening or ⩾300 cells·µL−1 during previous year before screening, baseline FEV1 <80% or FEV1/FVC <0.8 |
| Mepolizumab | SIRIUS (NCT01691508) (s.c. mepolizumab versus placebo to OCS+high-dose ICS and additional controller) (13) | 24 weeks | 135 | Daily OCS dose reduction | Higher proportion of patients with 90–100%reduction in mOCS dose (23% versus 11%) and 70–<90% reduction in mOCS (17% versus 8%). OR 2.39 for reduction in mOCS dose with mepolizumab | Reduction in annual AER versus placebo (1.44 versus 2.12) Improvement in ACQ-5 score from baseline by 0.52 points greater than in placebo (MCID, 0.5 points). Improvement in SGRQ score from baseline by 5.8 points greater than in placebo | 2014 | OCS-dependent severe eosinophilic asthma |
| Mepolizumab | MUSCA (NCT02281318) (s.c. mepolizumab versus placebo to OCS+high-dose ICS and additional controller) (14) | 24 weeks | 551 | Change in SGRQ score from baseline | Change from baseline in SGRQ total score mean improvement of -15.6 vs -7.9 with placebo. The treatment difference was -7.7 points (95% CI: -10.5 to -4.9; p < 0.0001) | Incidence of on-treatment adverse events (70% mepolizumab vs 74% placebo). Incidence of serious adverse events (5% mepolizumab vs 8% placebo). Common adverse events: headache, nasopharyngitis. | 2017 | Severe eosinophilic asthma |
| Reslizumab | NCT01287039; NCT01285323 (i.v. reslizumab versus placebo to medium-dose ICS) (15) | 52 weeks | 953 | Annual rate of clinical asthma exacerbations | Relative risk reduction in annual AER by 50–59%versus placebo | Improvement in AQLQ score from baseline by 0.27 points greater than in placebo at week 52 Improvement in ACQ-7 score from baseline by 0.25 points greater than in placebo at week 52 | 2015 | Moderate to severe asthma with ⩾1 exacerbation, blood eosinophil ⩾400 cells·µL−1 Patient on mOCS included |
| Reslizumab | NCT01270464 (i.v. reslizumab at 0.3 mg·kg−1 and 3 mg·kg−1 versus placebo to medium-dose ICS+blood eosinophil ⩾400 cells·µL−1) (16) | 16 weeks | 315 | Improved FEV₁ compared with placebo over 16 weeks in patients with persistent asthma and elevated blood eosinophil levels. | FEV₁ improved with absolute increases of 115 mL for the 0.3 mg/kg dose and 160 mL for the 3.0 mg/kg dose; FVC increased by 48 mL and 130 mL for the 0.3 mg/kg and 3.0 mg/kg doses, respectively | Improvement in mean ACQ score from baseline by 0.238–0.359 greater than in placebo (MCID, 0.5 points) Improvement in mean AQLQ score from baseline by 0.359 in 3 mg·kg−1 than in placebo (MCID, 0.5 points) | 2016 | Inadequately controlled asthma with poor ACQ-7, blood eosinophils ⩾400 cells·µL−1. Patients on mOCS excluded |
| Reslizumab | NCT01508936 (i.v. reslizumab versus placebo to medium-dose ICS) (17) | 16 weeks | 492 | Change in FEV₁ from baseline | No significant difference in FEV1 (255 mL versus 187 mL with between-group difference of 68 mL) | The mean ACQ-7 score improved by 0.195 points more than placebo, though this did not reach the minimal clinically important difference (MCID) threshold of 0.5 points. No significant differences were observed in FVC or SABA use when patients were not stratified by baseline eosinophil counts. | 2016 | Inadequately controlled asthma. Patients on mOCS were excluded |
| Benralizumab | SIROCCO ( NCT01928771) (s.c. benralizumab versus placebo to medium- to high-dose ICS+LABA±mOCS) (18) | 48 weeks | 1205 | Annual exacerbation rate ratio versus placebo | Reduction in annual AER with rate ratio of 0.55 every 4 weeks and rate ratio of 0.49 every 8 weeks over 48 weeks versus the placebo | Improvement in TASS from baseline at week 48 by 0.25 points greater (every 8 weeks) than in placebo. Improvement in mean ACQ-6 score from baseline by 0.29 points greater (every 8 weeks) than in placebo Improvement in mean AQLQ from baseline by 0.30 points greater (every 8 weeks) than in placebo | 2016 | Severe asthma with ⩾2 exacerbations, baseline FEV1 <80% at screening. Patients on mOCS included |
| Benralizumab | CALIMA (NCT01914757) (s.c. benralizumab versus placebo to medium- or high-dose ICS+LABA±mOCS) (19) | 56 weeks | 1306 | Annual exacerbation rate ratio versus placebo for patients receiving high-dosage ICS plus LABA with baseline blood eosinophils 300 cells/ μL or greater | Reduction in annual AER with rate ratio of 0.64 at every 4 weeks and rate ratio of 0.72 at every 8 weeks over 56 weeks versus the placebo | Improvement in TASS from baseline by 0.23 points with every 8 weeks dosing than in placebo Improvement in mean ACQ-6 from baseline by 0.19 points (every 4 weeks) and by 0.25 points (every 8 weeks) greater than in placebo Improvement in mean AQLQ (S)+12 from baseline by 0.24 points greater (every 8 weeks) than in placebo | 2016 | Severe uncontrolled asthma with ⩾2 exacerbations, baseline FEV1 <80% at screening. Patients on mOCS included |
| Benralizumab | ZONDA (NCT02075255) (s.c. benralizumab versus placebo to medium- to high-dose ICS+LABA+OCS) (20) | 28 weeks | 220 | The median percentage reduction in the final oral glucocorticoid (maintenance OCS) dose from baseline to week 28 | Benralizumab led to a 75% median mOCS dose reduction, versus 25% with placebo. ≥90% dose reduction was achieved by 33% (q4w) and 37% (q8w) of patients, compared to 12% with placebo. ≥75% reduction was seen in 53% (q4w) and 51% (q8w) vs. 20% with placebo. | A higher proportion of patients discontinued mOCS: 56% (q4w) and 52% (q8w) vs. 19% with placebo. Annual exacerbation rates (AER) were reduced with rate ratios of 0.45 (q4w) and 0.30 (q8w) vs. placebo ACQ-6 scores improved by 0.55 points more than placebo. AQLQ(S)+12 scores improved by 0.45 points over placebo. | 2017 | Severe asthma with blood eosinophil ⩾150 cells·µL−1, on mOCS for at least 6 months |
| Dupilumab | LIBERTY ASTHMA QUEST (s.c. dupilumab versus placebo to medium- or high-dose ICS+LABA, LAMA, anti-leukotriene and methylxanthines) (21) | 52 weeks | 1902 |  | Relative risk reduction in annual AER by 47.7% and 46% with 200 mg and 300 mg, respectively, versus placebo | Relative risk reduction in annual AER by 65.8% and 67.4% with 200 mg and 300 mg, respectively, versus placebo in subgroup with eosinophils ⩾300 Improvement in mean change in ACQ-5 score from baseline at week 52 by 0.39 and 0.22 points greater than in placebo with 200 mg and 300 mg dosing, respectively Improvement in mean change in AQLQ from baseline at week 52 by 0.29 and 0.26 points greater than in placebo, respectively, with 200 mg and 300 mg dosing | 2018 | Moderate to severe, uncontrolled asthma with ⩾1 exacerbations, baseline FEV1 ⩽80% pred at screening. Inclusion irrespective of baseline blood eosinophil count or biomarkers of T2 inflammation |
| Dupilumab | LIBERTY ASTHMA VENTURE  (s.c. dupilumab versus placebo to mOCS+high-dose ICS+LABA/LAMA) (22) | 24 weeks | 210 |  | Reduction in mOCS dose from baseline to week 24 was 70% versus 42% in placebo | Higher proportion of patients with at least 50% reduction in OCS dose at week 24 (80% in dupilumab versus 50% in placebo). Greater proportion of patients had mOCS dose reduction to <5 mg·day−1 in dupilumab (69% versus 33% in placebo) Cessation of mOCS at week 24 was 52% with dupilumab versus 29%with placebo | 2018 | Severe asthma on mOCS for at least previous 6 months, baseline FEV1 ⩽80% pred at screening. Inclusion irrespective of baseline blood eosinophil count or biomarkers of type 2 inflammation |
| Duplimab | VESTIGE (NCT04400318)  (add-on dupilumab 300 mg Q2W or placebo Q2W + medium-to-high dose ICS ± second controller) (23) | 24 weeks | 109 | Proportion of patients with FeNO <25 ppb at week 24 and percentage change from baseline in specific airway volume ([s]iVaw) at TLC at week 24 | Significantly more patients achieved FeNO <25 ppb with dupilumab vs placebo (57% vs 11%; OR 9.8, p<0.001). Numerical increase in [s]iVaw with dupilumab vs placebo (LSMD 21.8%, p=0.14; not significant) | Nominal improvements with dupilumab vs placebo: reduction in mucus plug score and volume, improved pre-/post-bronchodilator FEV₁, improved ACQ-7 score and improved airway resistance (R5-R20) and reactance area via FOT. Safety profile consistent with known dupilumab profile. | 2024 | Uncontrolled moderate-to-severe Type 2 asthma (blood eosinophils ≥300 cells/μL and FeNO ≥25 ppb) |
| Tezepelumab | PATHWAY (NCT02054130) (s.c. tezepelumab versus placebo to medium- or high-dose ICS+LABA). Tezepelumab dose-ranging trial (70 mg every 4 weeks, 210 mg every 4 weeks and 280 mg every 2 weeks) (24) | 52 weeks | 550 | Annualized asthma exacerbation | Relative risk reduction in annual AER at week 52 by 62–71% with the different doses of tezepelumab versus placebo | Longer time to first exacerbation in tezepelumab versus placebo. Improvement in mean change in ACQ-6 score from baseline at week 52 by 0.29 and 0.31 points greater than in placebo with medium and high dose, respectively Improvement in mean change in AQLQ(S) +12 from baseline at week 52 by 0.34 points greater than in placebo with high dose | 2017 | Uncontrolled moderate or severe asthma with ⩾2 exacerbations, baseline FEV1 40–80% pred at screening. Patients on mOCS included |
| Tezepelumab | NAVIGATOR (NCT03347279) (s.c. tezepelumab versus placebo to medium or high dose ICS±one controller medication±mOCS) (25) | 52 weeks | 1061 | Annualized asthma exacerbation | Reduction in annual AER with rate ratio of 0.44 at 52 weeks versus placebo Reduction in annual AER size in the subgroup with blood eosinophils ⩽300 cells·µL−1 with rate ratio of 0.59 at week 52 versus placebo | Improvement in mean change in ACQ-6 score from baseline at week 52 by 0.33 points greater with tezepelumab than in placebo. Improvement in mean change in AQLQ(S) +12 from baseline at week 52 by 0.34 points greater than in placebo | 2021 | Uncontrolled, moderate to severe asthma with ⩾2 exacerbations, baseline FEV1 <80% pred at screening .Patients on mOCS included |
| Tezepelumab | SOURCE  (NCT03406078) (s.c. tezepelumab versus placebo to medium- or high-dose ICS+LABA ±additional controller) (26) | 48 weeks | 150 | Proportion of patients achieving OCS dose reduction | Cumulative odds of categorised % reduction from baseline in daily mOCS dose at week 48 was similar between tezepelumab versus placebo (did not meet primary end-point) 54% of patients in tezepelumab and 46% of patients in the placebo reduced daily mOCS by 90–100% | Reduction in annual AER with rate ratio of 0.69 at 48 weeks versus placebo Median % reduction from baseline in daily mOCS dose at week 48 was 100% in tezepelumab versus 75% in the placebo | 2023 | Uncontrolled moderate or severe asthma with ⩾1 exacerbations on stable mOCS for at least 6 months, baseline FEV1 <80% pred at screening. Patients on mOCS included |
| Tezepelumab | PASSAGE (NCT05329194)  (Patients received tezepelumab 210 mg Q4W; single-arm, real-world study) (27) | 52 weeks | 208 | Annualized Asthma Exacerbation Rate (AAER) during treatment vs. baseline period | AAER reduced by 76% (95% CI: 69, 81) from 2.96 (baseline) to 0.71 (treatment) | Clinically meaningful improvements were observed, including increased pre-BD FEV₁ (+0.11 L overall; +0.19 L in patients with FEV₁ ≤80% predicted), alongside significant reductions in ACQ-6 (-1.1 points), AIRQ (-2.9 points), SGRQ (-22.8 points), and SNOT-22 scores. High responder rates (48-84%) were achieved across these measures, with a safety profile consistent with previous studies. | 2025 | Broad severe asthma phenotypes (BEC ≥/<300 cells/μL, allergic/non-allergic). Included underrepresented groups: Black/African American (17%), adolescents (5%), smokers (23%), comorbid mild-moderate COPD (13%) |

Abbreviations: AER: annual exacerbation rate; ACQ: Asthma Control Questionnaire; AQLQ: Asthma Quality of Life Questionnaire; AQLQ(S)+12: Asthma Quality of Life Questionnaire (standardised) for ⩾12 years of age; BDR: bronchodilator responsiveness; ER: emergency room; FENO: exhaled nitric oxide fraction; FEV1: forced expiratory volume in 1 s; FAO: fixed airflow obstruction; FVC: forced vital capacity; HR: hazard ratio; ICS: inhaled corticosteroid; LABA: long-acting beta agonist; LAMA: long-acting muscarinic antagonist; MCID: minimal clinical important difference; mOCS: maintenance oral corticosteroid; OCS: oral corticosteroid; OR: odds ratio; PEF: peak expiratory flow; QoL: Quality of Life; RAST: radioallergosorbent test; SABA: short-acting beta-agonist; SGRQ: St. George’s Respiratory Questionnaire; TASS: total asthma symptom severity score

Table S3 Biologic therapies for chronic rhinosinusitis with nasal polyps (CRSwNP) approved by EMA.

| **Medicine** | **Active substance** | **Mechanism of action** | **Biomarkers for response prediction** | **Approved patient population** | **Year of EMA approval** | **Dosing regimen** | **Safety profile / Common side effects** | **Formulation & presentation** | **Other indications** |
| --- | --- | --- | --- | --- | --- | --- | --- | --- | --- |
| **Dupixent (1)** | Dupilumab | Targets IL-4 receptor α, inhibiting IL-4 and IL-13 signaling pathways involved in type 2 inflammation. | Blood eosinophils, tissue eosinophils, FeNO, total IgE, T2 comorbidities (asthma, AD) | Adults with severe CRSwNP for whom therapy with systemic corticosteroids and/or surgery do not provide adequate disease control. | 2019 | Subcutaneous injection: initial dose of 300 mg, followed by 300 mg every 2 weeks. | Injection site reactions, eosinophilia, arthralgia. | Prefilled syringe or pen (300 mg). | Asthma, AD, eosinophilic esophagitis, prurigo nodularis, COPD. |
| **Xolair (1)** | Omalizumab | Binds to IgE, preventing its interaction with high-affinity IgE receptors on mast cells and basophils,  Reduces the amount of free IgE available to trigger the allergic cascade. | Total serum IgE, allergen sensitization, blood eosinophils | Adults (18 years and above) with severe CRSwNP for whom therapy with intranasal corticosteroids does not provide  adequate disease control. | 2020 | Subcutaneous injection: dose and frequency based on body weight and IgE level; typically every 2 or 4 weeks. | Headache, pyrexia, injection site reactions, upper abdominal pain. | Prefilled syringe or pen (75 mg or 150 mg). | Allergic asthma, chronic spontaneous urticaria. |
| **Nucala (2)** | Mepolizumab | Monoclonal antibody targeting IL-5, thereby reducing eosinophil production and survival. | Blood eosinophils, tissue eosinophils, history of systemic corticosteroid dependence; T2 comorbid asthma | Adults with severe CRSwNP and evidence of eosinophilic inflammation, uncontrolled with standard therapy. | 2021 | Subcutaneous injection: 100 mg every 4 weeks. | Lower respiratory tract infection, urinary tract infection, hypersensitivity reactions, pharyngitis, headache, nasal congestion, upper abdominal pain, eczema, back pain, arthralgia, injection site reactions, pyrexia. | Prefilled syringe or pen (100 mg). | Severe eosinophilic asthma, eosinophilic granulomatosis with polyangiitis (EGPA), hypereosinophilic syndrome (HES). |
| **Omyclo** (7) | Omalizumab | Binds to IgE, preventing its interaction with high-affinity IgE receptors on mast cells and basophils,  Reduces the amount of free IgE available to trigger the allergic cascade | Total serum IgE, allergen sensitization, blood eosinophils | Adults (18 years and above) with severe CRSwNP for whom therapy with intranasal corticosteroids does not provide  adequate disease control. | 2024 | Subcutaneous injection: dose and frequency based on body weight and IgE level; typically every 2 or 4 weeks. | Headache, pyrexia, injection site reactions, upper abdominal pain. | Prefilled syringe or pen (75 mg or 150 mg). | Allergic asthma, chronic spontaneous urticaria |

Abbreviations: AD: atopic dermatitis; COPD: chronic obstructive lung disease; CRSwNP: chronic rhinosinusitis with nasal polyps; EMA: European Medicines Agency; EGPA: eosinophilic granulomatosis with polyangiitis; FeNO: fractional exhaled nitric oxide; HES: hypereosinophilic syndrome; IgE: immunoglobulin E.

Notes regarding biologics available for CRSwNP:

- **Self-administration suitability:** all four biologics are designed for self-administration using prefilled syringes or pens, with appropriate patient training.
- **Pregnancy / Lactation data:** for all agents, data in pregnant or lactating women are limited. Animal studies have not shown harm, but human IgG is known to cross the placenta and is present in breast milk. Use during pregnancy or lactation should be considered only if clearly needed.
- **Storage & Handling:** all products should be stored refrigerated at 2°C to 8°C and protected from light. They can be kept at room temperature (≤25-30°C) for up to 14 days (Xolair for up to 48 hours Omyclo and Nucala for up to 7 days) (1,2,7)
- **Discontinuation:** consideration should be given to discontinuing biological treatment in patients who have shown no response after 24 weeks of treatment with Dupixent.
- **Immunogenicity data:** no evidence of ADA impact on pharmacokinetics, efficacy or safety.

Table S4 Major clinical studies on EMA-approved biologics in chronic rhinosinusitis with nasal polyps.

| **Biological** | **Study** | **Duration** | **Indication** | **Patients (n)** | **Primary endpoint** | **Primary outcome** | **Secondary outcome** | **Publication year** |
| --- | --- | --- | --- | --- | --- | --- | --- | --- |
| Omalizumab | POLYP 1 (NCT03280550) (s.c. Omalizumab (mg/kg) Q2W or Q4W or Placebo Q2W or Q4W + INCS (mometazone), as background treatment (28) | 24 weeks | Severe chronic rhinosinusitis with nasal polyps (CRSwNP) who had an inadequate response to intranasal corticosteroids | 138 | Change from baseline to week 24 in endoscopic NPS24 and mean daily NCS | The mean changes in NPS for omalizumab versus placebo from baseline at week 24 were:–1.08 versus + 0.06 (treatment arm difference, –1.14 [95% CI 5 –1.59 to –0.69; P < .0001])  The mean changes for omalizumab versus placebo in NCS from baseline to week 24 were: –0.89 versus –0.35 (treatment arm difference, –0.55 [95% CI 5 –0.84 to –0.25; P=0.0004]) | At week 24  SNOT-22 score (range, 0-110) Omalizumab –24.70 (2.01) vs –8.58 (2.08) Placebo; P<0.0001 UPSIT score (range, 0-40) Omalizumab 4.44 (0.84) vs 0.63 (0.90) Placebo; P=0.0024 TNSS (range, 0-12) Omalizumab –2.97 (0.33) vs –1.06 (0.34) Placebo P=0.0001 | 2020 |
| Omalizumab | POLYP 2 (NCT03280537) (s.c. Omalizumab (mg/kg) Q2W or Q4W or Placebo Q2W or Q4W + INCS (mometazone), as background treatment (28) | 24 weeks | Severe chronic rhinosinusitis with nasal polyps (CRSwNP) who had an inadequate response to intranasal corticosteroids | 127 | Change from baseline to week 24 in endoscopic NPS24 and mean daily NCS | The mean changes in NPS for omalizumab versus placebo from baseline at week 24 were:–0.90 versus –0.31 (treatment arm difference, –0.59 [95% CI 5 –1.05 to –0.12; P=0.0140])  The mean changes for omalizumab versus placebo in NCS from baseline to week 24 were:–0.70 versus –0.20 (treatment arm difference, –0.50 [95% CI 5 –0.80 to –0.19; P=0.0017]) | At week 24  SNOT-22 score (range, 0-110) Omalizumab –21.59 (2.25) vs –6.55 (2.19) Placebo; P<0.0001 UPSIT score (range, 0-40) Omalizumab 4.31 (0.83) vs 0.44 (0.81)) Placebo; P=0.0011 TNSS (range, 0-12) Omalizumab –2.53 (0.33) vs –0.44 (0.32)) Placebo; P=0.0001 | 2020 |
| Mepolizumab | SYNAPSE (NCT03085797) (mepolizumab at 100 mg s.c. or placebo once every 4 weeks (using a safety syringe), in addition to standard of care for 52 weeks) (29) | 52 weeks | Severe, recurrent CRSwNP, considered eligible for repeat sinus surgery | 407 | The coprimary endpoints were change from baseline in total endoscopic NPS at week 52 and in mean nasal obstruction VAS score during weeks 49–52 | Change from baseline in total endoscopic NPS at week 52: Mepolizumab –0·9 (1·90) vs Placebo –0·1 (1·46) ; P<0.0001. Change from baseline in nasal obstruction VAS score during weeks 49–52: Mepolizumab –4·2 (3·42) vs Placebo –2·5 (3·15) ; P<0.0001 | Proportion of patients having nasal surgery up to week 52 (time-to-first nasal surgery): Mepolizumab (9%) vs 23% Placebo; P=0.0032. Change from baseline in overall symptom VAS score during weeks 49–52: Mepolizumab –4·3 vs –2·5 Placebo; P=0.0032. Change from baseline in SNOT-22 total score at week 52: Mepolizumab–29·4 vs –15·7 Placebo; P=0.0032. Proportion of patients requiring systemic corticosteroids (≥1 course) for nasal polyps until week 52: Mepolizumab (25%) vs (37%) Placebo; P=0.020 | 2021 |
| Mepolizumab | MERIT (NCT04607005) (Mepolizumab 100 mg s.c. or placebo once every 4 weeks), in addition to standard of care (30) | 52 weeks | Severe chronic rhinosinusitis with nasal polyps (CRSwNP)/eosinophilic CRS (ECRS) who were eligible for repeat sinus surgery or were systemic corticosteroid users/medically unstable | 169 | Change from baseline in total endoscopic NP score at week 52 and the mean nasal obstruction VAS score during weeks 49–52. | Change from baseline in total endoscopic NP score at week 52: Mepolizumab -0.62 (0.16) vs Placebo -0.19 (0.16); treatment difference -0.43 (95% CI: -0.89, 0.03); p=0.067. Nasal obstruction VAS score during weeks 49–52: Mepolizumab -3.2 (0.34) vs Placebo -1.8 (0.33); treatment difference -1.43 (95% CI: -2.37, -0.50); p=0.003. | At week 52, mepolizumab showed greater improvements than placebo in multiple measures. SNOT-22 total score decreased by −18.27 versus −7.65 (difference −10.63; 95% CI: −18.68 to −2.57; p = 0.017). Nasal obstruction VAS improved by −3.2 versus −1.8 (difference −1.43; p = 0.003), and composite nasal symptom VAS by −2.64 versus −1.47 (difference −1.17; p = 0.005). LMK-CT score improved by −3.52 versus −1.88 (difference −1.63; p = 0.012), while loss-of-smell VAS decreased by −1.71 versus −0.89 (difference −0.82; p = 0.009). | 2024 |
| Benralizumab | OSTRO (NCT03401229) (30 mg benralizumab subcutaneously every 4 weeks for the first 3 doses then every 8 weeks (Q8W) thereafter or placebo + INCS background) (31) | 80 weeks | Severe, symptomatic CRSwNP despite standard intranasal corticosteroid treatment | 413 | NPS at week 40 (scale, 0-8). Biweekly mean NBS at week 40 (scale, 0-3) | NPS at week 40 (scale, 0-8) benralizumab -0.418 vs placebo 0.153; P <0.001. Biweekly mean NBS at week 40 (scale, 0-3): benralizumab -0.711 vs placebo -0.270; P 0.005 | SNOT-22 total score at week 40 (scale, 0-110): benralizumab -16.23 vs placebo -11.02; P 0.08. Time to first NP surgery and/or SCS use for NP: benralizumab 34.8% vs placebo 44.8%; P 0.07. Biweekly mean DSS score at week 40 (scale, 0-3): benralizumab -0.383 vs placebo -0.165; P 0.003. NPS at week 56 (scale, 0-8): benralizumab -0.361 vs placebo 0.114; P 0.005. Biweekly mean NBS at week 56 (scale, 0-3): benralizumab -0.703 vs placebo -0.416; P 0.003. SNOT-22 total score at week 56 (scale, 0-110): benralizumab -16.25 vs placebo -8.75; P 0.02 | 2021 |
| Dupilumab | LIBERTY NP SINUS-24 (NCT02912468) (s.c. dupilumab 300 mg every 2 weeks (q2w) or placebo q2w) (32) | 24 weeks | Severe CRSwNP and inadequate symptom control despite intranasal corticosteroids, systemic corticosteroids, or previous surgery | 276 | Coprimary endpoints were changes from baseline to week 24 in nasal polyp score (NPS), nasal congestion or obstruction | LSM difference in NPS of dupilumab treatment versus placebo was -2.06 (95% CI -2.43 to -1.69; P<0.0001) Difference in nasal congestion or obstruction score was -0.89 (-1.07 to -0.71; P<0.0001) | **At week 24**  *(LS mean difference vs placebo)* Lund–Mackay CT score (scale 0–24) –7·44 (–8.35 to –6.53; p<0.0001) Total symptom score (scale 0–9) –2.61 (–3.04 to –2.17; p<0·0001). Smell test score (UPSIT;scale 0–40) 10.56 (8.79 to 12.34;p<0.0001). Loss-of-smell score (scale 0–3) –1·12 (–1·31 to –0·93;p<0·0001). SNOT-22 score (scale 0–110) –21·12 (–25·17 to –17·06; p<0·0001) | 2019 |
| Dupilumab | LIBERTY NP SINUS-52 (NCT02898454) (s.c.dupilumab 300 mg q2w for52 weeks; dupilumab 300 mg SC q2w for 24 weeks, then 300 mg s.c. every 4 weeks for 28 weeks; or placebo q2w for 52 weeks) (32) | 52 weeks | Severe CRSwNP and inadequate symptom control despite intranasal corticosteroids, systemic corticosteroids, or previous surgery | 448 | Coprimary endpoints were changes from baseline to week 24 in nasal polyp score (NPS), nasal congestion or obstruction | LSM difference in NPS of dupilumab treatment versus placebo was -1.80 (-2.10 to -1.51; P<0.0001). Difference in nasal congestion or obstruction score was -0.87 (-1.03 to -0.71; p<0.0001) | **At week 24** *(LS mean difference vs placebo).* Lund–Mackay CT score (scale 0–24) –5·13 (–5·80 to –4·46;p<0.0001). Total symptom score (scale 0–9) –2·44 (–2·87 to –2·02;p<0·0001). Smell test score (UPSIT;scale 0–40) 10·52 (8·98 to 12·07; p<0·0001). Loss-of-smell score (scale 0–3) –0·98 (–1·15 to –0·81;p<0·0001). SNOT-22 score (scale 0–110) –17·36 (–20·87 to –13·85; p<0·0001)  **At week 52**  *(LS mean difference vs placebo).* Bilateral nasal polyp score (scale 0–8) –2.40 (–2.77 to –2.02;p<0·0001). Nasal congestion or obstruction score (scale 0–3) –0.98 (–1.17 to –0.79;p<0.0001). SNOT-22 score (scale 0–110) –20.96(–25.03 to –16.89;p<0.0001) | 2019 |

Abbreviations: INCS: Intranasal corticosteroid;LSM: Least-squares mean;NPS: Nasal Polyp Score;SNOT-22: Sino-Nasal Outcome Test-22;TNSS: Total Nasal Symptom Score;UPSIT: University of Pennsylvania Smell Identification Test

# Reference list

1. Xolair: EPAR – Product Information [Internet]. European Medicines Agency. https://www.ema.europa.eu/en/medicines/human/EPAR/xolair [Accessed May 26, 2025]

2. Nucala: EPAR – Product Information [Internet]. European Medicines Agency. https://www.ema.europa.eu/en/medicines/human/EPAR/nucala [Accessed May 26, 2025]

3. Cinqaero: EPAR – Product information [Internet]. European Medicines Agency. https://www.ema.europa.eu/en/medicines/human/EPAR/cinqaero [Accessed May 26, 2025]

4. Fasenra: EPAR – Product information [Internet]. European Medicines Agency. https://www.ema.europa.eu/en/medicines/human/EPAR/fasenra [Accessed May 26, 2025]

5. Dupixent: EPAR – Product information [Internet]. European Medicines Agency. https://www.ema.europa.eu/en/medicines/human/EPAR/dupixent [Accessed May 26, 2025]

6. Tezspire: EPAR – Product information [Internet]. European Medicines Agency. https://www.ema.europa.eu/en/medicines/human/EPAR/tezspire [Accessed May 26, 2025]

7. Omlyclo: EPAR – Product information [Internet]. European Medicines Agency. https://www.ema.europa.eu/en/medicines/human/EPAR/omlyclo [Accessed May 26, 2025]

8. Humbert M, Beasley R, Ayres J, Slavin R, Hébert J, Bousquet J, Beeh K-M, Ramos S, Canonica GW, Hedgecock S, et al. Benefits of omalizumab as add-on therapy in patients with severe persistent asthma who are inadequately controlled despite best available therapy (GINA 2002 step 4 treatment): INNOVATE. *Allergy* (2005) 60:309–316. doi: 10.1111/j.1398-9995.2004.00772.x

9. Hanania NA, Alpan O, Hamilos DL, Condemi JJ, Reyes-Rivera I, Zhu J, Rosen KE, Eisner MD, Wong DA, Busse W. Omalizumab in severe allergic asthma inadequately controlled with standard therapy: a randomized trial. *Annals of internal medicine* (2011) 154:573–582. doi: 10.7326/0003-4819-154-9-201105030-00002

10. Hanania NA, Fortis S, Haselkorn T, Gupta S, Mumneh N, Yoo B, Holweg CTJ, Chipps BE. Omalizumab in Asthma with Fixed Airway Obstruction: Post Hoc Analysis of EXTRA. *The Journal of Allergy and Clinical Immunology: In Practice* (2022) 10:222–228. doi: 10.1016/j.jaip.2021.08.006

11. Pavord ID, Korn S, Howarth P, Bleecker ER, Buhl R, Keene ON, Ortega H, Chanez P. Mepolizumab for severe eosinophilic asthma (DREAM): a multicentre, double-blind, placebo-controlled trial. *Lancet (London, England)* (2012) 380:651–659. doi: 10.1016/S0140-6736(12)60988-X

12. Ortega HG, Liu MC, Pavord ID, Brusselle GG, FitzGerald JM, Chetta A, Humbert M, Katz LE, Keene ON, Yancey SW, et al. Mepolizumab treatment in patients with severe eosinophilic asthma. *The New England journal of medicine* (2014) 371:1198–1207. doi: 10.1056/NEJMoa1403290

13. Bel EH, Wenzel SE, Thompson PJ, Prazma CM, Keene ON, Yancey SW, Ortega HG, Pavord ID. Oral glucocorticoid-sparing effect of mepolizumab in eosinophilic asthma. *The New England journal of medicine* (2014) 371:1189–1197. doi: 10.1056/NEJMoa1403291

14. Chupp GL, Bradford ES, Albers FC, Bratton DJ, Wang-Jairaj J, Nelsen LM, Trevor JL, Magnan A, Ten Brinke A. Efficacy of mepolizumab add-on therapy on health-related quality of life and markers of asthma control in severe eosinophilic asthma (MUSCA): a randomised, double-blind, placebo-controlled, parallel-group, multicentre, phase 3b trial. *The Lancet Respiratory medicine* (2017) 5:390–400. doi: 10.1016/S2213-2600(17)30125-X

15. Castro M, Zangrilli J, Wechsler ME, Bateman ED, Brusselle GG, Bardin P, Murphy K, Maspero JF, O’Brien C, Korn S. Reslizumab for inadequately controlled asthma with elevated blood eosinophil counts: results from two multicentre, parallel, double-blind, randomised, placebo-controlled, phase 3 trials. *The Lancet Respiratory medicine* (2015) 3:355–366. doi: 10.1016/S2213-2600(15)00042-9

16. Bjermer L, Lemiere C, Maspero J, Weiss S, Zangrilli J, Germinaro M. Reslizumab for Inadequately Controlled Asthma With Elevated Blood Eosinophil Levels: A Randomized Phase 3 Study. *Chest* (2016) 150:789–798. doi: 10.1016/j.chest.2016.03.032

17. Corren J, Weinstein S, Janka L, Zangrilli J, Garin M. Phase 3 Study of Reslizumab in Patients With Poorly Controlled Asthma: Effects Across a Broad Range of Eosinophil Counts. *Chest* (2016) 150:799–810. doi: 10.1016/j.chest.2016.03.018

18. Bleecker ER, FitzGerald JM, Chanez P, Papi A, Weinstein SF, Barker P, Sproule S, Gilmartin G, Aurivillius M, Werkström V, et al. Efficacy and safety of benralizumab for patients with severe asthma uncontrolled with high-dosage inhaled corticosteroids and long-acting β(2)-agonists (SIROCCO): a randomised, multicentre, placebo-controlled phase 3 trial. *Lancet (London, England)* (2016) 388:2115–2127. doi: 10.1016/S0140-6736(16)31324-1

19. FitzGerald JM, Bleecker ER, Nair P, Korn S, Ohta K, Lommatzsch M, Ferguson GT, Busse WW, Barker P, Sproule S, et al. Benralizumab, an anti-interleukin-5 receptor α monoclonal antibody, as add-on treatment for patients with severe, uncontrolled, eosinophilic asthma (CALIMA): a randomised, double-blind, placebo-controlled phase 3 trial. *Lancet (London, England)* (2016) 388:2128–2141. doi: 10.1016/S0140-6736(16)31322-8

20. Nair P, Wenzel S, Rabe KF, Bourdin A, Lugogo NL, Kuna P, Barker P, Sproule S, Ponnarambil S, Goldman M. Oral Glucocorticoid-Sparing Effect of Benralizumab in Severe Asthma. *The New England journal of medicine* (2017) 376:2448–2458. doi: 10.1056/NEJMoa1703501

21. Castro M, Corren J, Pavord ID, Maspero J, Wenzel S, Rabe KF, Busse WW, Ford L, Sher L, FitzGerald JM, et al. Dupilumab Efficacy and Safety in Moderate-to-Severe Uncontrolled Asthma. *The New England journal of medicine* (2018) 378:2486–2496. doi: 10.1056/NEJMoa1804092

22. Rabe KF, Nair P, Brusselle G, Maspero JF, Castro M, Sher L, Zhu H, Hamilton JD, Swanson BN, Khan A, et al. Efficacy and Safety of Dupilumab in Glucocorticoid-Dependent Severe Asthma. *The New England journal of medicine* (2018) 378:2475–2485. doi: 10.1056/NEJMoa1804093

23. Castro M, Papi A, Porsbjerg C, Lugogo NL, Brightling CE, González-Barcala F-J, Bourdin A, Ostrovskyy M, Staevska M, Chou P-C, et al. Effect of dupilumab on exhaled nitric oxide, mucus plugs, and functional respiratory imaging in patients with type 2 asthma (VESTIGE): a randomised, double-blind, placebo-controlled, phase 4 trial. *The Lancet Respiratory Medicine* (2025) 13:208–220. doi: 10.1016/S2213-2600(24)00362-X

24. Corren J, Parnes JR, Wang L, Mo M, Roseti SL, Griffiths JM, van der Merwe R. Tezepelumab in Adults with Uncontrolled Asthma. *The New England journal of medicine* (2017) 377:936–946. doi: 10.1056/NEJMoa1704064

25. Menzies-Gow A, Corren J, Bourdin A, Chupp G, Israel E, Wechsler ME, Brightling CE, Griffiths JM, Hellqvist Å, Bowen K, et al. Tezepelumab in Adults and Adolescents with Severe, Uncontrolled Asthma. *The New England journal of medicine* (2021) 384:1800–1809. doi: 10.1056/NEJMoa2034975

26. Wechsler ME, Menzies-Gow A, Brightling CE, Kuna P, Korn S, Welte T, Griffiths JM, Sałapa K, Hellqvist Å, Almqvist G, et al. Evaluation of the oral corticosteroid-sparing effect of tezepelumab in adults with oral corticosteroid-dependent asthma (SOURCE): a randomised, placebo-controlled, phase 3 study. *The Lancet Respiratory medicine* (2022) 10:650–660. doi: 10.1016/S2213-2600(21)00537-3

27. Lugogo NL, Akuthota P, Sumino K, Mathur SK, Burnette AF, Lindsley AW, Llanos J-P, Marchese C, Ambrose CS, Emmanuel B. Effectiveness and Safety of Tezepelumab in a Diverse Population of US Patients with Severe Asthma: Initial Results of the PASSAGE Study. *Adv Ther* (2025) 42:3334–3353. doi: 10.1007/s12325-025-03231-6

28. Gevaert P, Omachi TA, Corren J, Mullol J, Han J, Lee SE, Kaufman D, Ligueros-Saylan M, Howard M, Zhu R, et al. Efficacy and safety of omalizumab in nasal polyposis: 2 randomized phase 3 trials. *The Journal of allergy and clinical immunology* (2020) 146:595–605. doi: 10.1016/j.jaci.2020.05.032

29. Han JK, Bachert C, Fokkens W, Desrosiers M, Wagenmann M, Lee SE, Smith SG, Martin N, Mayer B, Yancey SW, et al. Mepolizumab for chronic rhinosinusitis with nasal polyps (SYNAPSE): a randomised, double-blind, placebo-controlled, phase 3 trial. *The Lancet Respiratory medicine* (2021) 9:1141–1153. doi: 10.1016/S2213-2600(21)00097-7

30. Fujieda F, Wang C, Yoshikawa M, Asako M, Suzaki I, Bachert C, Han JK, Fuller A, Baylis L, Su L, et al. Mepolizumab in CRSwNP/ECRS and NP: the phase III randomised MERIT trial in Japan, China, and Russia. *Rhin* (2024) 0:0–0. doi: 10.4193/Rhin24.156

31. Bachert C, Han JK, Desrosiers MY, Gevaert P, Heffler E, Hopkins C, Tversky JR, Barker P, Cohen D, Emson C, et al. Efficacy and safety of benralizumab in chronic rhinosinusitis with nasal polyps: A randomized, placebo-controlled trial. *The Journal of allergy and clinical immunology* (2022) 149:1309-1317.e12. doi: 10.1016/j.jaci.2021.08.030

32. Bachert C, Han JK, Desrosiers M, Hellings PW, Amin N, Lee SE, Mullol J, Greos LS, Bosso J V, Laidlaw TM, et al. Efficacy and safety of dupilumab in patients with severe chronic rhinosinusitis with nasal polyps (LIBERTY NP SINUS-24 and LIBERTY NP SINUS-52): results from two multicentre, randomised, double-blind, placebo-controlled, parallel-group phase 3 trials. *Lancet (London, England)* (2019) 394:1638–1650. doi: 10.1016/S0140-6736(19)31881-1
